# Retromer stabilization results in neuroprotection in a model of Amyotrophic Lateral Sclerosis

**DOI:** 10.1038/s41467-020-17524-7

**Published:** 2020-07-31

**Authors:** Luca Muzio, Riccardo Sirtori, Davide Gornati, Simona Eleuteri, Andrea Fossaghi, Diego Brancaccio, Leonardo Manzoni, Linda Ottoboni, Luca De Feo, Angelo Quattrini, Eloise Mastrangelo, Luca Sorrentino, Emanuele Scalone, Giancarlo Comi, Luciana Marinelli, Nilo Riva, Mario Milani, Pierfausto Seneci, Gianvito Martino

**Affiliations:** 1 INSPE—Institute of Experimental Neurology, San Raffaele Scientific Institute, Milano, Italy; 2 Department of Chemistry, University of Milan, Milano, Italy; 3 Department of Pharmacy, University of Naples “Federico II”, Naples, Italy; 4 Institute of Molecular Science and Technology (ISTM), CNR, Milan, Italy; 5 Institute of Biophysics (IBF), CNR, Milan, Italy

**Keywords:** Protein aggregation, Organelles, Amyotrophic lateral sclerosis, Motor neuron

## Abstract

Amyotrophic Lateral Sclerosis (ALS) is a fatal disease characterized by the degeneration of upper and lower motor neurons (MNs). We find a significant reduction of the retromer complex subunit VPS35 in iPSCs-derived MNs from ALS patients, in MNs from ALS post mortem explants and in MNs from SOD1G93A mice. Being the retromer involved in trafficking of hydrolases, a pathological hallmark in ALS, we design, synthesize and characterize an array of retromer stabilizers based on bis-guanylhydrazones connected by a 1,3-phenyl ring linker. We select compound 2a as a potent and bioavailable interactor of VPS35-VPS29. Indeed, while increasing retromer stability in ALS mice, compound 2a attenuates locomotion impairment and increases MNs survival. Moreover, compound 2a increases VPS35 in iPSCs-derived MNs and shows brain bioavailability. Our results clearly suggest the retromer as a valuable druggable target in ALS.

## Abstract (web summary)

ALS is a neurodegenerative disease characterized by loss of motor neurons. Here, the authors showed that reduced levels of the VSP35 subunit in the retromer complex is a conserved ALS feature and identified a new lead compound increasing retromer stability ameliorating the disease phenotype.

## Introduction

Amyotrophic Lateral Sclerosis (ALS) is a devastating disorder, featured by a progressive degeneration of brain and spinal cord (SC) motor neurons (MNs). Clinical outcomes involve muscle weakness, movement defects, speech disturbances, dysphasia, and eventually death by respiratory failure^1^. The pathobiology of ALS is not completely understood, although the presence of cytoplasmic inclusions of protein aggregates in neurons is a key hallmark of ALS pathophysiology^2^.

Most ALS patients (90%) are sporadic, while only ≈10% display a positive family history (fALS). Around 20% of fALS patients show mutations in the copper/zinc superoxide dismutase (*SOD1*) gene^3^. The ALS-linked G93A mutation of *SOD1* allowed the establishment of a mouse model, featuring MN degeneration in the SC^4^. Mutant SOD1 proteins misfold, filling MNs with toxic aggregates, which impinge on their survival^5^. Unlike proliferating cells, which dilute aggregated proteins at each round of cell division, neurons degrade these aggregates only through the ubiquitin-proteasome system, or the autophagy-lysosomal pathway^6^.

Lysosomal health is crucial for the degradation of dysfunctional proteins and in particular for the clearance of autophagic vacuoles. An impaired lysosomal system contributes to autophagy stress, accumulation of damaged mitochondria, and restricts clearance of proteins aggregates. Remarkably, lysosome impairment is among the earliest pathological events that affect MNs in G93A mice^7^.

Delivery of proteolytic enzymes to lysosomes needs the coordinated action of multiple protein regulators, among which the highly conserved retromer complex^8^. The retromer complex was initially described for its ability to sort plasma membrane proteins from endosomes to the trans-Golgi network, or back to the cell surface. Two subcomplexes assemble in the cytoplasm to produce the retromer complex. The former is a trimeric complex composed by vacuolar protein sorting (VPS) 35, 29, and 26 that assemble into the cargo recognition core (CRC) complex^9^. The latter consists of sorting-nexin (SNX) proteins, either containing phox-homology (PX) and bin/amphiphysin/rvs (BAR) domains, or only the former one^10–13^. The retromer recycles specific cargos, such as Vps10/Sortilin protein family members^14^ or the cation-independent mannose 6-phosphate receptor (CI-MPR)^15^. The latter is involved in the delivery of proteolytic enzymes to lysosomes. A point mutation in VPS35 (D620N) is causative of an autosomal dominant form of Parkinson’s disease (PD)^16,17^ and affects early steps of autophagosome formation^18^. VPS35 deficiency alters the distribution of lysosome-associated membrane glycoprotein 2a (Lamp2a) in neurons^19^.

Pharmacological tools, increasing the interaction between members of the CRC complex, increase retromer stability and enhance retromer-mediated trafficking of cargos. The isothiourea chaperone R55/**1** stabilizes the VPS35-VPS29 interaction, increases retromer levels in vitro, and decreases pathogenic processing of APP in cells^20^.

We describe a substantial reduction of CRC proteins in MNs of G93A mice; a similar VPS35 downregulation in SCs from ALS patients and in inducible pluripotent stem cells (iPSCs)-derived MNs. We design, synthetize and characterize a small array of bis-guanylhydrazones. Such retromer stabilizers possess good in vivo bioavailability, potency, and stability. We selected phenyl-1,3-bis-guanylhydrazone **2a** for extended in vitro and in vivo characterization in ALS models.

## Results

### CRC proteins are downregulated in G93A mice

**Fig. 1:**
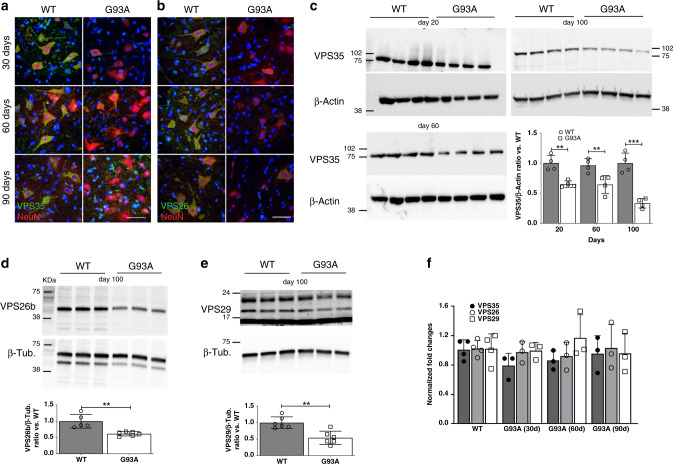
CRC proteins are downregulated in G93A MNs. **a** Maximal projections of confocal stacks of VPS35^+^NeuN^+^ putative MNs in the ventral horn of lumbar SCs from WT and G93A mice (30, 60 and 90 days, *n* = 4 biologically independent mice/time point). Adjacent sections labelled for VPS26 and NeuN are shown in **b**, (30, 60 and 90 days, *n* = 4 biologically independent mice/time point). **c** WBs for VPS35 and β-Actin in lumbar SCs protein extracts from WT and G93A mice at 20, 60, and 100 days (*n* = 4 biologically independent mice/time point). The histogram shows quantifications (mean ± SD) of normalized VPS35 levels (ratio versus WT), (WT vs. G93A day20: *p* = 0.007; day 60: *p* = 0.0015; day 90: *p* < 0.0001). **d** Representative WB for VPS26b and β-Tubulin in SCs from WT and G93A mice at day 100. The histogram shows the quantification (mean ± SD) of normalized VPS26b levels (ratio versus WT, *n* = 5 biologically independent WT mice and *n* = 6 biologically independent G93A mice, *p* = 0.002). **e** Representative WB for VPS29 levels in SCs from WT and G93A mice at day 100. The histogram shows the quantification (mean ± SD) of normalized VPS29 levels (ratio versus WT, *n* = 6 biologically independent mice/group, *p* = 0.0019). **f** quantification of *Vps35*, *Vps26*, and *Vps29* mRNAs by real-time PCR (ratio versus WT) in lumbar SCs from WT (sampled at day 60, *n* = 4 biologically independent mice) and G93A mice (30, 60, and 90 days, *n* = 3 biologically independent mice/time point). Two-way ANOVA followed by Bonferroni multiple comparisons test was used to analyze data of **c**. Two-tailed Student’s *t*-test was used to determine the statistical significance in **d** and **e**. One-way ANOVA followed by Tukey's Multiple Comparison test was used to analyze data of **f**. **p < 0.01; ***p < 0.001. Scale bar 80 µm.

A loss of Cathepsin D (CSTD) along with endo-lysosomal deficits affects MNs of G93A mice^7^. As these alterations could implicate retromer functionality^21^, we studied CRC proteins in SCs from G93A mice. VPS35 immunoreactivity in cultured neurons is organized in puncta that localize throughout the cell soma, dendrites, and axons^9,22,23^. Accordingly, immunofluorescence (IF) analysis of VPS35^+^ puncta showed their clustering in the soma and cytoplasmic bundles of ventral horn wild-type (WT) MNs (Fig. 1a and Supplementary Fig. 1a). VPS35 immunoreactivity was substantially attenuated in MNs of asymptomatic and symptomatic G93A mice (Fig. 1a and Supplementary Fig. 1b, c). Similarly, we observed attenuated VPS26 immunoreactivity in parallel SC sections (Fig. 1b and Supplementary Fig. 1d–f). We next assayed the levels of VPS35, VPS29, and the VPS26b isoform highly expressed in the CNS^24^ by western blotting lumbar SC extracts from G93A mice. We observed a significant reduction of VPS35 in asymptomatic and symptomatic G93A mice, as well as a reduction of VPS26b and VPS29 in symptomatic G93A mice (Fig. 1c–e and Supplementary Fig. 1g, h). These results confirm that a loss of any of the CRC components leads to instability and rapid degradation of the remaining proteins^25^. Conversely, we did not find alterations of VPS35 and VPS26 immunoreactivities in NeuN^+^ cells sampled in the motor cortex of G93A mice (Supplementary Fig. 2a–f). We next assayed *Vps35*, *Vps26*, and *Vps29* mRNAs levels in lumbar SC extracts by real-time PCR. Despite the reduction of VPS protein levels, their mRNA levels did not drop significantly when compared with WT controls, suggesting that their reduction was not caused by transcriptional inhibition (Fig. 1f).

### Design and synthesis of new pharmacological chaperones

R55/**1** binds to a characterized hot spot at the interface between VPS35 and VPS29^20^. We replaced the 2,5-disubstituted thiophene scaffold in R55/**1** with a phenyl ring. Phenyl and thiophene rings are bio-isosters^26^, and the former offers more synthetic versatility, i.e., can be substituted for structure activity relationship (SAR) acquisition. We substituted isothioureas with guanylhydrazones, as the latter groups show good stability and a pKa range closer to neutrality due to the delocalization of their charge on three N atoms (favored azine form vs. disfavored hydrazone form, 4.5 ≤ ΔGT ≤ 6.5 Kcal/mol^27^, Supplementary Fig. 3). The pKa range of guanylhydrazones should ensure a higher permeability through lipophilic bio-membranes, due to an estimated ≈16% free base at 37 °C and pH = 7.4^28^. Their presence in drugs^29^ endowed with blood-brain barrier (BBB) permeability^30^ supports our choice.

**Fig. 2:**
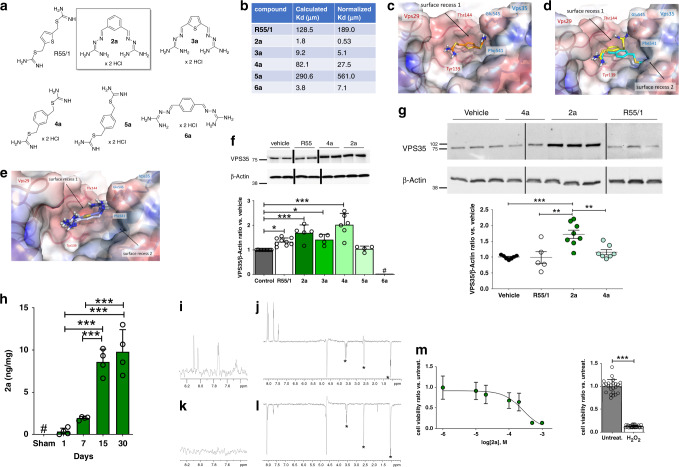
Structure and binding modes for R55/1 and compounds 2a–6a. **a** Standard R55/**1**, compounds bearing one (**3a**–**5a**) or two modifications (**2a**, **6a**), **b** Calculated and normalized Kd values for compounds **2a**–**6a**. **c** R55/**1** (orange carbons atoms) positioned near a small cavity (surface recess 1) outlined by Tyr139 and Thr144 in VPS29, and Phe541 in VPS35. **d** 1,3-meta substituted **4a** (yellow carbon atoms) better fits into surface recess 1; 1,4-meta substituted **5a** (cyan carbon atoms) repositions an isothiourea group in surface recess 2, with a predicted loss of affinity. **e** docking poses of 1,3-phenyl bis-isothiourea **3a** (violet carbon atoms) and 1,3-phenyl bis-guanylhydrazone **2a** (grey carbon atoms) in their putative binding site. **f** representative WB for VPS35 and β-Actin in treated Neuro2a cells (10 μM, 48 h, 3 × 10^5^ cells/well). Dividing black lines mark lanes cropped from the same filter. The histogram shows quantifications (control: *n* = 11 independent wells; R55/**1**: *n* = 9 independent wells; **2a**: *n* = 5 independent wells; **3a**: *n* = 4 independent wells; **4a**: *n* = 6 independent wells; **5a**: *n* = 4 independent wells; **6a**: *n* = 4 independent wells). Control vs. R55/1: *p* = 0.036, control vs. **2a**: *p* < 0.0001, control vs. **3a**: *p* = 0.048, control vs. **4a**: *p* < 0.0001. **g** representative WB for VPS35 and β-Actin in SCs extracts from vehicle-, R55/**1**-, **2a**- and **4a**-treated (10 mg/kg for 7 days) C57BL/6J mice. Dividing black lines mark lanes cropped from parallel filters. The histogram shows quantifications (mean ratio ± SD, vehicle: *n* = 7 independent mice; R55/**1**: *n* = 5 independent mice; **2a**: *n* = 8 independent mice; **4a**: *n* = 7 independent mice. Vehicle vs. **2a** *p* < 0.0001, R55/**1** vs. **2a** *p* = 0.002, **2a** vs.**4a** *p* = 0.0012). **h** Brain uptake of compound 2a in C57BL/6J mice injected (10 mg/kg) with **2a** (1, 7, 15, and 30 days, *n* = 4 independent mice/time point, mean ± SD, *p* < 0.0001). **i**–**l** STD-NMR (**i**, **k**) and WL (**j**, **l**) spectra of **2a** (500 μM). Spectra (i) and (j) are in presence of VPS29-VPS35 complex (10 μM),  spectra (**k**) and (**l**) are controls. Stars indicate the proton resonances of the buffer. **m** Neuro2a cell viability (mean ± SD, reported as ratio versus controls) in presence of increasing amounts of **2a** (1–1000 µM, 48 h, 4 × 10^4^ cells/well, *n* = 24 independent wells/group, three experiments). Nonlinear fitting shows an LD_50_ of 257.9 µM. Controls were established by H_2_O_2_ treatment (200 µM for 48 h, *n* = 24 independent wells/group, untreated vs. H_2_O_2_: *p* < 0.0001). One-way ANOVA followed by Tukey's Multiple Comparison test was used to analyze **f**, **g**, and **h** plots. Two-tailed Student’s *t*-test was used to analyze data from **m**. *p < 0.05, **p < 0.01, ***p < 0.001.

Six compounds were then studied in silico for their putative affinity for the retromer complex. Standard thiophene bis-isothiourea R55/**1**^20^, and compounds bearing one (**3a**–**5a**) or both described modifications (**2a**, **6a**) (Fig. 2a) were studied in silico for their putative affinity for the retromer complex. Their docking site was set at the interface between VPS35 and VPS29 (PDB code: 2R17), where R55/**1** binding was revealed by point mutation^20^. We determined the interaction energy between each compound (best pose) and the VPS35-VPS29 interface (calculated Kd). A normalized Kd took into account its recurrence in 40 simulation runs using a sigmoid function/multiplication factor (MF), varying between 2 (lowest recurrence, i.e., 1 in 40 poses) and 0.1 (highest recurrence, i.e., 40 in 40 poses). The function is neutral (MF = 1) for a 50% recurrence (20 in 40 poses), and favors/penalizes compounds with high/low recurrent poses (Supplementary Fig. 4a). Figure 2b lists calculated and normalized Kd values for standard R55/**1** and compounds **2a**–**6a**, which show limited differences. We docked R55/**1** (orange yellow in Fig. 2c), obtaining a calculated Kd = 128.5 µM and a normalized Kd = 189.0 µM (15/40 poses). Its best docking pose suggested a thiophene–benzene ring replacement filling a small surface recess surrounded by the Phe541 residue in VPS35, and by Tyr139 and Thr144 residues in VPS29 (surface recess 1, Fig. 2c). Therefore, we modelled either a 1,3-(meta, compound **4a**, yellow in Fig. 2d) or a 1,4-phenyl bis-isothiourea (para, compound **5a**, light blue in Fig. 2d). In silico docking suggested that meta-substitution should preserve the favorable geometry of R55/**1**, slightly improving the interaction with surface recess 1 (calculated Kd = 82.1 µM for 4a) with good recurrence (normalized Kd = 27.5 µM-29/40 poses). Para substitution showed a reduced interaction (calculated Kd = 290.6 µM) with low recurrence (normalized Kd = 561 µM-7/40 poses) through repositioning of an isothiourea group in **5a** toward another surface cavity (surface recess 2, Fig. 2d), and the loss of electrostatic interactions with Glu545. We then replaced isothioureas with two meta-guanylhydrazones (compound **3a**, violet in Fig. 2e), showing a pose similar to R55/1, with improved affinity (calculated Kd = 9.2 µM) and good recurrence (normalized Kd = 5.1 µM-25/40 poses). When phenyl and 1,3 guanylhydrazones were introduced in compound **2a** (gray in Fig. 2e and Supplementary Fig. 4b, e–g), it displayed a favorable orientation with the phenyl ring filling surface recess 1, further increasing affinity for the putative binding site (calculated Kd = 1.8 µM) with high recurrence (normalized Kd = 0.53 µM-35/40 poses). The good interaction of phenyl-1,4 guanylhydrazone (compound **6a**, calculated Kd = 3.8 µM) was penalized by its low recurrence (normalized Kd = 7.1 µM-9/40 poses).

The predicted partition coefficient (logP—bioavailability) and pKa values (neutral/charged ratio at physiological pH) were calculated for compounds **2a**–**6a**, and standard R55/**1**. LogP values around 2.1 were suggested for isothioureas **1**, **4a**, and **5a**, while values around 0 were suggested for guanylhydrazones **2a**, **3a**, and **6a**. Both values are aligned with Lipinski rules^31^, supporting bioavailability for both compound families. Conversely, predicted pKa values were lower for guanylhydrazones **2a**, **3a,** and **6a** (pKa1 ≈ 7.5, pKa2 ≈ 8.1) than for isothioureas **1**, **4a**, and **5a** (pKa1 ≈ 10.2, pKa2 ≈ 10.8), suggesting a higher abundance of neutral, BBB-permeable guanylhydrazones at physiological pH.

We synthesized compounds **2a**–**6a** through short, high-yielding strategies: isothioureas **4a** and **5a** from dichloromethylbenzenes and isothiourea (Supplementary Fig. 4c); phenyl guanylhydrazones **2a**, **3a**, and **6a** from aryl dialdehydes and aminoguanidine (Supplementary Fig. 4d). We incubated Neuro2a cells with compounds **2a**–**6a** (10 µM) or with standard R55/**1** (10 µM) for 48 h. Protein extracts were collected, separated by SDS-PAGE and immunoblotted for VPS35 and the housekeeping gene β-Actin. We observed limited cytotoxicity in cells receiving compound **6a** that prevented the measurement of their VPS35 levels. As expected, VPS35 levels increased in R55/1-treated cells^20^. Similarly, compounds **2a**, **3a**, and **4a** increased VPS35 levels, while compound **5a** did not (Fig. 2f). Compound **2a** displays the lowest Kd value among molecules **2a**–**6a**, and we checked whether it could increase VPS26b and VPS29 levels. Accordingly, VPS26b and VPS29 proteins were significantly increased by compound **2a** in Neuro2a cells after treatment for 48 h (Supplementary Fig. 5a).

### In vivo delivery of compound 2a to the brain

Compounds **2a**, **4a**, and standard R55/**1** (10 mg/Kg) were intraperitoneally injected in C57BL6J mice daily. After 1 week, mice were sacrificed, saline perfused to avoid CNS contamination by compounds in blood circulation, and SC protein extracts were assayed by WB. While treatment with isothiourea **4a** slightly, but not significantly, increased VPS35 levels, phenyl-1,3-guanylhydrazone **2a** significantly increased VPS35 levels in SC extracts (Fig. 2g). Pharmacokinetic analysis of C57BL6J brains receiving daily intraperitoneal injections of compound **2a** confirmed its access to the CNS (Fig. 2h). Therefore, phenyl-1,3-bis-guanylhydrazone **2a** was selected as an early lead.

### NMR studies

We used a ligand-based NMR approach to localize the binding between compound **2a** and the VPS29-VPS35 heterodimer. These techniques provide binding information without requiring any isotopic labeling of the proteins. Particularly, Saturation Transfer Difference NMR (STD-NMR)^32^ and WaterLogsy-NMR (WL-NMR)^33^ experiments were acquired in buffer solutions containing a large excess of compound **2a** (500μM) with respect to VPS29-VPS35 (10 μM). Both STD-NMR and WL-NMR focus on the NMR signals of the ligand and utilize the magnetization transfer by the Nuclear Overhauser Effect (NOE) between protein and ligand. STD-NMR experiments are carried out by subtraction of a 1D ^1^H spectrum where the protein protons are selectively irradiated (on-resonance spectrum) from a spectrum in which the protein is not saturated (off-resonance spectrum). The resulting STD spectrum only shows ligand proton signals in contact with the macromolecule. The STD-NMR spectrum (Fig. 2i) shows the aromatic signals of compound **2a** in presence of the VPS29-VPS35 heterodimer, strongly indicating an interaction between the ligand and the protein complex.

WL-NMR entails magnetization transfer to the ligand from either bulk water via a macromolecule, and/or the exchangeable protons and the water molecules present in the binding site. Positive peaks for proton resonances of the ligand in the WL-NMR spectrum are observed for binders in presence of the protein. Otherwise, negative peaks will indicate that a putative ligand does not interact with the macromolecule. In the WL-NMR spectrum (Fig. 2j) the aromatic signals of compound **2a** in presence of the VPS29-VPS35 heterodimer show positive peaks, while proton resonances of the buffer (highlighted with stars in Fig. 2j, l) show a negative phase, indicating that **2a** binds to the protein complex. As negative control, STD-NMR and WL-NMR experiments were performed for compound **2a** in absence of the heterodimer. The absence of proton signals in the aromatic zone and the presence of negative signals in STD-NMR (Fig. 2k) and WL-NMR (Fig. 2l) spectra, respectively, confirms the binding between compound **2a** and the VPS29-VPS35 heterodimer. Unfortunately, STD- and WL-NMR competition experiments between **2a** and R55/**1** could not be performed since the latter compound was not stable in our experimental conditions.

### Compound 2a increases VPS35 levels

We next measured VPS35 degradation rates by a cycloheximide (CHX) chase assay. Neuro2a cells received vehicle or compound **2a** (10 µM) for 24 h, then CHX was added and VPS35 levels were measured by WB. As reported^34^, we observed that vehicle-treated cells exhibited a drop of VPS35 levels (∼40%) after 8 h of CHX treatment. Conversely, pretreating cells with compound **2a** protected VPS35 from degradation (Supplementary Fig. 6a, b).

Compound **2a**, as R55/**1**^20^, did not influence the transcription levels of CRC genes in Neuro2a cells, as determined by measuring *Vps35*, *Vps26*, and *Vps29* mRNAs levels by real-time PCR (Supplementary Fig. 6c).

We assessed the cytotoxicity of compound **2a**, measuring survival of Neuro2a cells exposed to increasing **2a** concentrations. The calculated LD_50_ was ∼260 µM (Fig. 2m), a higher value than the concentration needed to stabilize the retromer in vitro. We next checked for toxic effects of compound **2a** in primary neuronal cultures coupled with Micro Electrode Array (MEA) devices. Mouse cortical neurons (E16.5) were plated on MEAs, kept in culture for 14 days to develop firing activity and recorded in presence of **2a**. Low concentrations of compound **2a** did not alter neuronal firing, while we observed a slight reduction of spikes only when neurons were incubated with 100 μM of **2a** (Supplementary Fig. 7a–c). These results indicate a wide therapeutic window for compound **2a**, considering the low μM concentration needed in vitro to increase VPS35 levels.

### Compound 2a counteracts SOD1^G93A^-mediated cytotoxicity

**Fig. 3:**
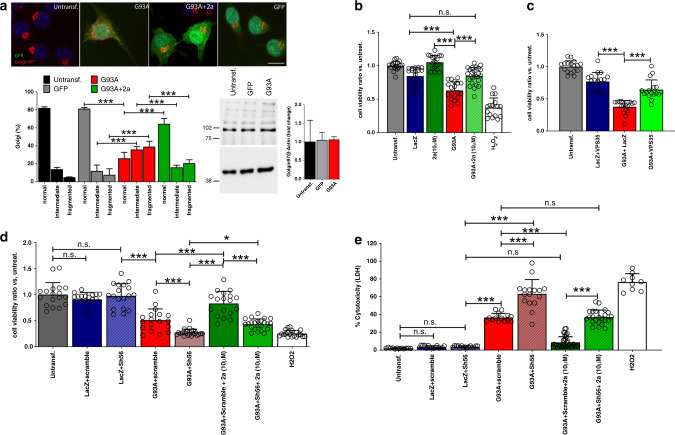
Compound 2a counteracts G93A-mediated Golgi fragmentation. **a** merged confocal stacks for GFP and Golgin97 in untransfected-; pcDNA3.1(+)SOD1^G93A^ (1 µg/well)-pCAAG-GFP (0.2 µg/well)-; pcDNA3.1(+)SOD1^G93A^ (1 µg/well)-pCAAG-GFP (0.2 µg/well) + **2a** (10 µM)- and pCAAG-GFP (1.2 µg/well)-transfected Neuro2a (4 × 10^4^ cells/well). Morphological analysis of Golgi in normal, intermediate or fragmented was done in nontransfected cells (*n* = 83 independent cells), G93A-GFP (*n* = 88 independent cells), G93A-GFP+ **2a** (*n* = 84 independent cells), and GFP (*n* = 109 independent cells). The histogram in panel **a** shows Golgi subclasses (% of the total number of Golgi constructs, means ± SD) examined over four independent experiments, *p* < 0.0001 for all comparisons. Protein lysates from nontransfected and transfected Neuro2a cells (3 × 10^5^ cells/well, pcDNA3.1(+)SOD1^G93A^ (2 µg/well)-pCAAG-GFP (0.5 µg/well) or with pCAAG-GFP (2 µg/well) were immunoblotted with antibodies against Golgin97 and β-Actin. The histogram shows quantifications (mean fold changes ± SD versus untreated cells, *n* = 3 wells/group). **b** Neuro2a cell viabilities (mean ratio ± SD compared to untransfected cells, *n* = 19 independent wells) upon the following treatments: pCMV-LacZ (1.25µg/well, *n* = 18 independent wells), pcDNA3.1(+)SOD1^G93A^ (1.25 µg/well, *n* = 18 independent wells), pcDNA3.1(+)SOD1^G93A^ (1.25 µg/well) + **2a** (10 µM, *n* = 24 independent wells), and **2a** (10 µM, *n* = 18 independent wells), (4 × 10^4^cells/well). Controls were obtained treating Neuro2A cells with H_2_O_2_ (200 µM, *n* = 16 independent wells). Data are examined over three independent experiments (LacZ vs. G93A+**2a** *p* > 0.99; *p* < 0.0001 for the other comparisons). **c** Neuro2a cell viabilities (mean ratio ± SD versus untreated cells) upon the following treatments: pCMV-LacZ+pcCDNA3.1-VPS35 (1 µg/well + 0.25 µg/well); pcDNA3.1(+)SOD1^G93A^+pCMV-LacZ (1 µg/well + 0.25 µg/well) and pcDNA3.1(+)SOD1^G93A^+pcCDNA3.1(+)VPS35 (1 µg/well + 0.25 µg/well), (4 × 10^4^ cells/well); *n* = 17 biologically independent wells/group examined over three independent experiments (*p* < 0.0001 for all comparisons). **d** Neuro2a cell viabilities (mean ratio ± SD versus untreated Neuro2a, 4 × 10^4^ cells/well, *n* = 18 independent wells) upon the following treatments: pCMV-LacZ (1 µg/well, *n* = 18 independent wells) with scramble or with Sh56 plasmids (0.25 µg/well each, *n* = 18 independent wells); pcDNA3.1(+)SOD1^G93A^ (1 µg/well) with scramble (*n* = 18 independent wells) or with Sh56 plasmids (0.25 µg/well, *n* = 23 independent wells) and with or without compound **2a** (10 µM, *n* = 18 independent wells); data are examined over three independent experiments (Untrans. vs LacZ+scramble *p* = 0.72, Untrans. vs. LacZ+Sh56 *p* = 0.99, G93A+Sh56 vs. G93A+Sh56+**2a** *p* = 0.036, *p* < 0.0001 for the other comparisons). **e** LDH assay in Neuro2a (4 × 10^4^ cells/well) receiving treatments and conditions as in panel **d**. Cytotoxicity (mean % ± SD) was calculated by measuring amounts of LDH released in the supernatant (Untransf.: *n* = 12 independent wells, LacZ+scramble and LacZ+Sh56: *n* = 15 independent wells; G93A+scramble: *n* = 14 independent wells; G93A+Sh56: *n* = 16 independent wells; G93A+scramble+**2a**: *n* = 36 independent wells; G93A+Sh56+**2a**: *n* = 23 independent wells, H_2_O_2_: *n* = 9 independent wells); data are examined over three independent experiments (Untrans. vs LacZ+scramble *p* = 0.99, Untrans. vs. LacZ+Sh56 *p* = 0.98 G93A+scramble vs. G93A+Sh56+**2a** *p* = 0.73, *p* < 0.0001 for the other comparisons). Two-way ANOVA followed by Bonferroni multiple comparisons test was used to analyze data of panel **a**. One-way ANOVA followed by Tukey's Multiple Comparison test was used to analyze data of **b**–**e**. *p < 0.05, ***p < 0.001, n.s. not significant. Scale bar in **a**, 10 µm.

The Golgi apparatus is affected in MNs from ALS patients and in cells overexpressing mutant SOD1 forms^35–37^. We transiently transfected Neuro2a cells with plasmids encoding *SOD1*^*G93A*^ before treatment with compound **2a**. Confocal analysis of cells labelled for trans-Golgi network marker Golgin97 revealed that many SOD1^G93A+^ cells displayed trans-Golgi fragmentation. Compound **2a** significantly reverted this phenotype (Fig. 3a). We next determined the viability of Neuro2a cells transfected with *SOD1*^*G93A*^ and exposed to compound **2a** using a colorimetric assay. Controls cells received *LacZ* plasmids. Compound **2a** (10 μM, 48 h) did not affect cell survival (Fig. 2l and Fig. 3b). We observed a slight, but not significant, reduction of cell viability in *LacZ*-transfected Neuro2a cells (Fig. 3b). When *SOD1*^*G93A*^ was overexpressed, cell viability was greatly reduced. Notably, cell viability was restored in *SOD1*^*G93A*^-transfected and compound **2a**-treated Neuro2a cells (Fig. 3b). The increased cell viability provided by compound **2a** could result from unknown, nonspecific off-target effects. Therefore, we used plasmids encoding *VPS35* to increase/stabilize the retromer complex. Overexpression of *VPS35* did not affect Neuro2a cell viability, while *SOD1*^*G93A*^ greatly affected their survival (Fig. 3c). However, this phenotype was reverted co-transfecting *VPS35* and *SOD1*^*G93A*^ plasmids (Fig. 3c).

Having observed a marked improvement of viability in *SOD1*^*G93A*^-transfected Neuro2a cells with VPS35 gain of function, we investigated the effect of VPS35 loss of function. We transfected Neuro2a cells with RNAi plasmids encoding a siRNA duplex for VPS35, to knockdown VPS35 and disrupt the retromer assembly^38^. The Sh56 plasmid significantly reduced VPS35 protein and mRNA levels and VPS35 immunoreactivity in cells, when compared with a scramble plasmid (Supplementary Fig. 8a–e). Downregulation of VPS35 by Sh56-encoding plasmids did not affect Neuro2a cell survival (Fig. 3d). As previously shown, cells receiving *SOD1*^*G93A*^ plasmids displayed a significant reduction of cell viability. By including Sh56 plasmids in the transfection mix, we worsened this phenotype (Fig. 3d). Notably, incubation of *SOD1*^*G93A*^-Sh56 double transfected cells with compound **2a** modestly increased cell viability, confirming that compound 2a counteracts SOD1^G93A^-mediated cytotoxicity by increasing VPS35 levels and retromer functionality. We next assayed cytotoxicity by measuring lactate dehydrogenase (LDH) release with a LDH-Glo™ cytotoxicity assay. Cells were treated with plasmids encoding *SOD1*^*G93A*^ and the Sh56-RNAi sequence with or without compound **2a**, while supernatants were used to measure LDH contents. Overexpression of *SOD1*^*G93A*^ increased LDH release by Neuro2aA cells, when compared with control cells (Fig. 3e). Compound **2a** significantly reduced LDH release in cells transfected with *SOD1*^*G93A*^, while LDH increased when cells received *SOD1*^*G93A*^ and Sh56 plasmids. Interestingly, high LDH release was observed in Neuro2a cells transfected with *SOD1*^*G93A*^ and Sh56 plasmids and treated with compound **2a**, further confirming that **2a** counteracts SOD1^G93A^-mediated cytotoxicity by targeting the retromer (Fig. 3e).

### Compound 2a ameliorates the phenotype of G93A mice

**Fig. 4:**
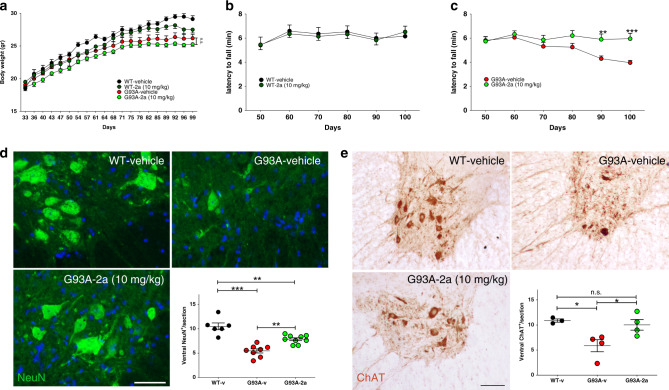
Compound 2a increases locomotion of G93A mice and MNs survival. **a** Body weight curves measured every 3 days in vehicle-treated WT mice (*n* = 10 independent mice); vehicle-treated G93A mice (*n* = 15 independent mice); compound **2a**-treated WT mice (*n* = 10 independent mice) and compound **2a**-treated G93A mice (*n* = 16 independent mice). Data (mean ± SEM) are examined over two independent experiments. **b** Latency to fall on the rotating bar of a rotarod apparatus in vehicle- and compound **2a**-treated WT mice (*n* = 10 independent mice/group from two independent experiments). Data are reported as mean ± SEM. **c** Latency to fall in vehicle- and in compound **2a**-treated G93A mice (*n* = 15 independent mice and *n* = 16 independent mice, respectively). Data (mean ± SEM) are examined from two independent experiments (G93A-v vs. G93A-**2a** day 90 *p* = 0.0041, day 100 *p* = 0.0006). **d** Lumbar SCs sections from vehicle-treated WT mice, vehicle-treated G93A mice and compound **2a**-treated G93A mice (10 mg/kg, sampled at day 100) labelled for NeuN. Quantifications were done in the ventral horn of the SC scoring MNs (WT-v: *n* = 6 independent mice, G93A-v: *n* = 9 independent mice, G93A-2a: *n* = 9 independent mice; lines show means ± SEM and are examined from two independent experiments). WT-v vs. G93A-v *p* < 0.0001, WT-v vs. G93A-**2a** *p* = 0.0017, G93A-v vs. G93A-**2a** *p* = 0.002. **e** IHC for ChAT on lumbar SCs sections. Quantifications of ChAT^+^/section in the ventral horn of the SC are shown in the histogram (WT-v: *n* = 3 independent mice, G93A-v: *n* = 4 independent mice, G93A-2a: *n* = 4 independent mice; lines show means ± SEM and are examined from one experiment). WT-v vs. G93A-v *p* = 0.026, WT-v vs. G93A-**2a** *p* = 0.84, G93A-v vs. G93A-**2a** *p* = 0.043. Two-tailed Student’s *t*-test was used to determine the statistical significance of data plotted in **a**. Two-way ANOVA followed by Bonferroni multiple comparisons test was used to analyze data from **b** and **c**. One-way ANOVA followed by Tukey's Multiple Comparison test was used to analyze data from **d** and **e**. *p < 0.05, **p < 0.01, ***p < 0.001, n.s. not significant. Scale bar 50 µm.

**Fig. 5:**
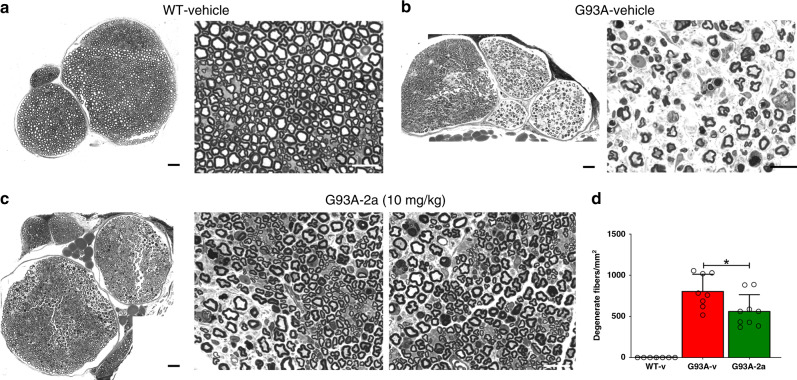
Compound 2a attenuates sciatic nerve degeneration in G93A mice. **a** Low and high magnifications of a sciatic nerve cross section from a vehicle-treated WT mouse. **b** Low and high magnifications of a sciatic nerve cross section from a vehicle-treated G93A mouse. **c** Low and high magnifications of a sciatic nerve cross section from a compound **2a** (10 mg/kg)-treated G93A mouse (day 100). Quantifications (means + SD) of degenerating fibers/mm^2^ scored in the entire cross section of nerves are shown in **d**, and data are examined over two independent experiments (WT *n* = 7 independent mice, G93A-vehicle *n* = 8 independent mice, G93A-compound **2a** *n* = 9 independent mice).(G93A-v vs. G93A-**2a**: *p* = 0.02. One-way ANOVA followed by Tukey's Multiple Comparison test was used to analyze data. *p < 0.05. Scale bars 50 µm for low-magnification images and 20 µm for high-magnification images.

We administered compound **2a** (10 mg/kg, i.p.) daily to G93A and WT littermates between day 30 and day 100. We observed a slight, but not significant reduction of body weight in both WT and G93A mice (Fig. 4a). We did not observe alterations of locomotion performances in WT mice treated with compound **2a** (Fig. 4b). We also did not observe distress or undesirable pain. Strikingly, the progression of motor deficits in G93A mice was significantly slowed down by compound **2a** (Fig. 4c). Histopathological analysis of lumbar SCs showed more MNs in ventral horns of SCs from compound **2a**-treated G93A mice than from vehicle-treated G93A mice (Fig. 4d, e). We next assessed the pathological degeneration of fibers in sciatic nerves of G93A mice treated with compound **2a**. We observed axonal degeneration, with a substantial reduction of myelinated nerve fibers in vehicle-treated G93A mice (Fig. 5a, b)^39^. Conversely, mice treated with compound **2a** showed a ∼30% reduction of degenerating fibers (Fig. 5c, d). Using Luxol Fast Blue (LFB) to label lipoproteins of the myelin sheath^40^, we analyzed myelination. Compound **2a**-treated G93A mice displayed a substantial reduction of demyelination when compared with vehicle-treated G93A mice (Supplementary Fig. 9a–d). Parallel sections, labelled for myelin basic protein (MBP) and medium neurofilaments (NF-M), showed deranged myelin in vehicle-treated G93A mice while compound **2a** treatment preserved the integrity of myelin in G93A mice (Supplementary Fig. 9e).

Cell-secreted SOD1^G93A^ in the extracellular space activates microglia by interaction with CD14^41,42^. Therefore, we analyzed microglia/macrophages in SCs scoring Iba1^+^ cells and measured CD14 expression levels in G93A mice. Neither CD14 levels nor the total number of Iba1^+^ cells changed in compound **2a**-treated G93A mice when compared with vehicle-treated mice (Supplementary Fig. 10a–c). Our finding supports earlier reports of a rescue of behavioral deficits and pathological outcomes in 3XTg AD mice treated with an AAV-VPS35 vector, without functional alterations of the microglia/macrophage cell population^43,44^.

### Compound 2a increases VPS35/VPS26 levels in G93A mice

**Fig. 6:**
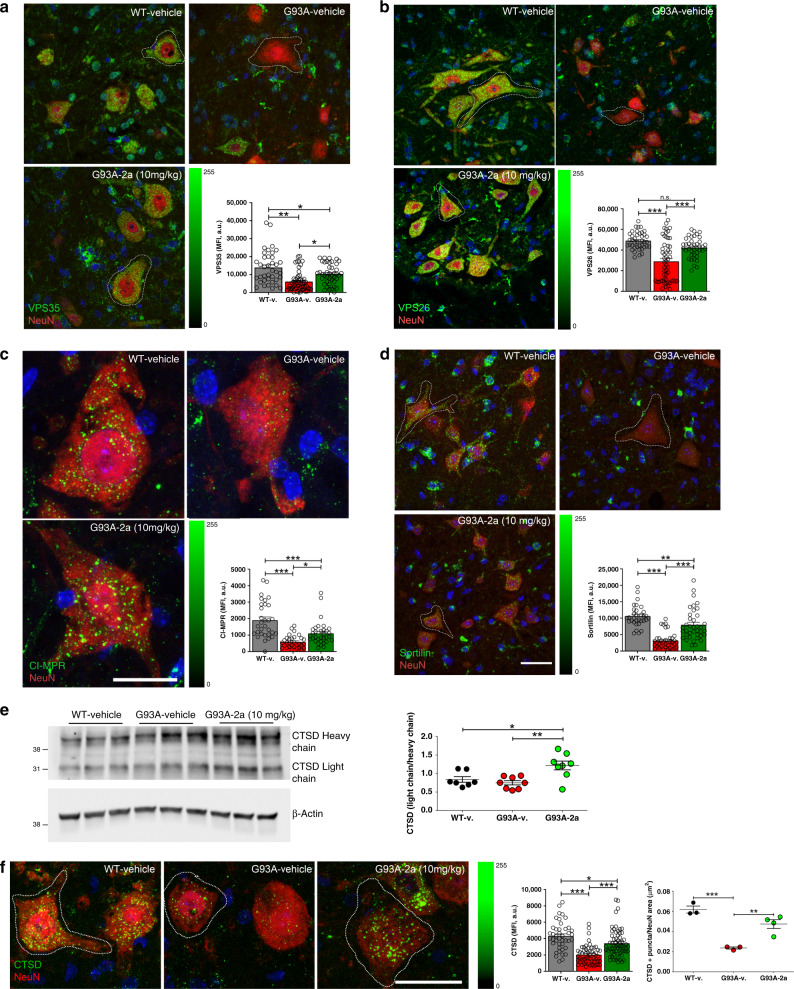
Compound 2a increases retromer proteins in G93A mice. (**a**–**d**, **f**) maximum projections of confocal stacks from lumbar SCs (ventral horn) of vehicle-treated WT, vehicle-treated G93A and compound 2a (10 mg/kg, day 100)-treated G93A mice (*n* = 3 independent mice/group) labelled for: VPS35/NeuN (**a**), VPS26/NeuN (**b**), CI-MPR/NeuN (**c**), Sortilin/NeuN (**d**), and CTSD/NeuN (**f**). Averaged fluorescence intensities (MFI) in gated NeuN^+^ MNs (WT-v: *n* = 38 independent MNs, G93A-v: *n* = 54 independent MNs, G93A-**2a**: *n* = 42 independent MNs, data were examined from three independent experiments), of VPS35 (Alexafluor 488) are reported as mean arbitrary units (a.u. ± SEM) in Panel a (WT-v vs. G93A-v *p* = 0.002, WT-v vs. G93A-**2a** *p* = 0.046, G93A-v vs. G93A-**2a** *p* = 0.011). Averaged MFI (a.u. ± SEM) of VPS26 (Alexafluor 488) quantified in gated NeuN^+^ MNs (WT-v: *n* = 40 independent MNs, G93A-v: *n* = 54 independent MNs, G93A-**2a**: *n* = 40 independent MNs, data are examined from three independent experiments) are shown in **b** (G93A-v vs. G93A-**2a** *p* = 0.09, other comparisons *p* < 0.0001). Averaged MFI of CI-MPR (Alexafluor 488, a.u. ± SEM) in NeuN^+^ MNs (WT-v: *n* = 31 independent MNs, G93A-v: *n* = 31 independent MNs, G93A-**2a**: *n* = 33 independent MNs, data are examined from three independent experiments) are shown in **c** (G93A-v vs. G93A-**2a** *p* = 0.026, other comparisons *p* < 0.0001). Averaged Sortilin MFI levels (Alexafluor 488 a.u. ± SEM) quantified in gated NeuN^+^ MNs (WT-v: *n* = 33 independent MNs, G93A-v: *n* = 37 independent MNs, G93A-**2a**: *n* = 38 independent MNs, data are examined from three independent experiments) are shown in **d**. Epi-fluorescence scales are shown on the right side of **a**–**d** (WT-v vs. G93A-**2a** *p* = 0.009, other comparisons *p* < 0.0001). **e** Representative WB for CTSD (heavy (46–50 KDa) and light (28–30 KDa chains). Normalization of proteins load was done by β-Actin. The histogram in **e** shows quantifications (WT-v: *n* = 7 independent mice, G93A-v: *n* = 8 independent mice, G93A-**2a**: *n* = 8 independent mice, data are examined from two independent experiments; lines show mean + SEM). WT-v vs. G93A-**2a** *p* = 0.02, G93A-v vs. G93A-**2a** *p* = 0.0032. **f** Averaged CTSD MFI levels (Alexafluor 488, a.u. ± SEM) in gated NeuN^+^ MNs (WT-v: *n* = 42 independent MNs, G93A-v: *n* = 54 independent MNs, G93A-**2a**: *n* = 63 independent MNs, data are examined from three independent experiments). The epi-fluorescence scale is shown in **f** (WT-v vs. G93A-**2a** *p* = 0.011, other comparisons *p* < 0.0001). Numbers of CTSD puncta normalized for the area of NeuN^+^ MNs are shown in the histogram (WT-v: *n* = 3 independent mice, G93A-v: *n* = 3 independent mice, G93A-**2a**: *n* = 4 independent mice, data are examined from two independent experiments, lines show mean + SEM). (WT-v vs. G93A-v *p* = 0.0005, G93A-v vs. G93A-**2a** *p* = 0.006. One-way ANOVA followed by Tukey's Multiple Comparison test was used to analyze data in **a**–**f**. *p < 0.05, **p < 0.01, ***p < 0.001. Scale bars 30 µm for **a**, **b**, **d**, and **f**; 25 µm for **c**.

Standard R55/**1** increases VPS35 and VPS26 levels, and retromer functionality in cultured cells^20^. We asked whether compound **2a** could do the same in G93A MNs, using confocal microscopy to quantify VPS35 fluorescence levels in MNs gated in the ventral horn of lumbar SCs. We observed a significant reduction of VPS35 mean fluorescence intensity (MFI) levels in vehicle-treated G93A mice. In contrast, treatment with compound **2a** significantly increased such levels in MNs of G93A mice (Fig. 6a). We next assayed VPS26 MFI levels on parallel sections. As expected, MFI levels were reduced in vehicle-treated G93A mice, while they were significantly increased in compound **2a**-treated mice (Fig. 6b). WBs of lumbar SC extracts confirmed the ability of **2a** to increase VPS35 and VPS26b levels in G93A mice (Supplementary Fig. 11a, b).

We next asked whether increasing VPS35/26 protein levels is sufficient to increase retromer functionality. CI-MPR is unstable in VPS26-knockout cells^21^, and we observed a substantial reduction of CI-MPR MFI levels in MNs of vehicle-treated G93A mice compared with WT litters (Fig. 6c). However, MFI levels significantly increased in compound **2a**-treated G93A MNs (Fig. 6c). Sortilin encodes a receptor belonging to the Vps10 family involved in Frontotemporal Dementia (FTD)^45^, which shares functional homologies with MPRs and is another retromer cargo^46^. Sortilin MFI levels were significantly reduced in MNs of vehicle-treated G93A mice, while significantly increased by compound **2a** (Fig. 6d).

We next investigated Cathepsin D (CTSD) expression and distribution in G93A mice, since its trafficking involves the retromer complex^21^. In humans, CTSD maturation starts with a pre-pro-CTSD then converted to pro-CTSD (52 kDa) in the endoplasmic reticulum. Recycling of pro-CTSD and its downstream migration to acidic compartments involves CI-MPR and the retromer pathway^21^. Pro-CTSD is further processed to an intermediate form in late endosomes (48 kDa), and finally converted to mature CTSD (34 kDa) in lysosomes^47^. VPS35 inactivation^48^ or VPS26 knockdown^21^ affect CTSD maturation. We scored CTSD levels in lumbar SC extracts from vehicle-treated WT and vehicle- or **2a**-treated G93A mice. We observed a slight, but not significant reduction of the mature CTSD/pro-CTSD ratio in SC extracts of vehicle-treated G93A mice compared with WT litters. Possibly, we did not see a reduction of mature CTSD in total protein lysates because glial cells of G93A mice express high amounts of mature CTSD at day 100^7^ (Fig. 6e). However, levels of mature CTSD significantly increased in compound **2a**-treated G93A mice (Fig. 6e). We next analyzed CTSD levels and CTSD^+^ puncta in MNs by confocal microscopy, observing their significant reduction in MNs of vehicle-treated G93A mice that was restored by treatment with compound **2a** (Fig. 6f). These data suggest that retromer stabilization mediated by compound **2a** restores Sortilin and CI-MPR expression levels in ALS mice and improves CTSD maturation, thus restoring lysosome health in ALS mice^7,49^.

### Compound 2a increases lysosomal homeostasis in G93A mice

**Fig. 7:**
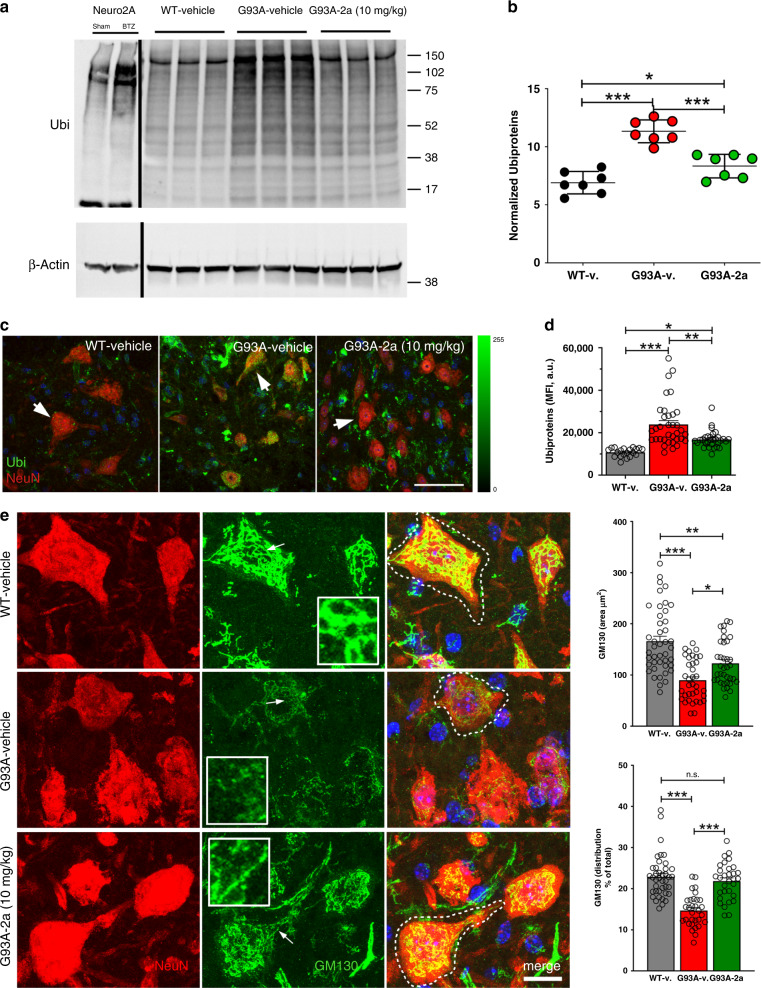
Compound 2a reduces protein ubiquitination in G93A mice. **a** Representative WB for poly-ubiquitinated proteins in experimental controls: Neuro2a cells (3 × 10^5^ cells/well) treated with sham or with Bortezomib (10 nM, BTZ); and in lumbar SC extracts from vehicle-treated WT mice, vehicle-treated G93A mice and compound **2a**-treated G93A mice (10 mg/kg, day 100). Normalization of proteins load was done by β-Actin. Dividing black lines show lanes cropped from independent filters. **b** Collective densities of lanes, normalized for β-Actin, were used for quantification (*n* = 7 independent mice/group data are examined from three independent experiments, lines show means + SD). WT-v vs G93A-**2a**: *p* = 0.013, *p* < 0.0001 for the other comparisons. **c** Maximum projections of confocal stacks from lumbar SCs sections of vehicle-treated WT, vehicle-treated G93A and compound **2a**-treated G93A mice (10 mg/kg, sampled at day 100) labelled for ubiquitin (Ubi) and NeuN (*n* = 3 independent mice/group). Averaged fluorescence intensities (MFI, lines show means + SEM) in gated Neun^+^ MNs of Ubi (Alexafluor 488) are reported in **d** (arrows indicate representative cells; WT-v: *n* = 20 independent MNs, G93A-v: *n* = 35 independent MNs, G93A-**2a**: *n* = 31 independent MNs, data are examined from three independent experiments; WT-v vs. G93A-**2a**: *p* = 0.014, *p* < 0.0001 for the other comparisons). The epi-fluorescence scale is shown in right side. **e** Maximum projections of confocal stacks (step of 0.4 µm) of MNs labelled for NeuN and GM130. Arrows in each panel indicate regions showed at high magnification in insets. Quantifications (means ± SEM) of GM130 area are shown in upper **e**, (WT-v: *n* = 43 independent MNs, G93A-v: *n* = 36 independent MNs, G93A-**2a**: *n* = 36 independent MNs, data are examined from three independent experiments; G93A-v vs. G93A-**2a**: *p* = 0.0018; *p* < 0.0001 for the other comparisons). Quantifications of GM130 distribution (mean% of the cell area ± SEM) are shown in lower panel **e**, (WT-v: *n* = 40 independent MNs, G93A-v: *n* = 31 independent MNs, G93A-**2a**: *n* = 31 independent MNs, data are examined from three independent experiments; WT-v vs. G93A-**2a**: *p* = 0.63, *p* < 0.0001 for the other comparisons). One-way ANOVA followed by Tukey's Multiple Comparison test was used to analyze data of **b**–**e**. *p < 0.05, **p < 0.01, ***p < 0.001, n.s. not significant. Scale bar 50 µm in **c** and 20 µm in **e**.

Ubiquitinated inclusions accumulate in MNs from sALS and fALS patients^50^, and in ALS mice^51^. We characterized poly-ubiquitinated proteins in lumbar SCs protein extracts by 10% denaturating PAGE. Protein extracts from Neuro2a cells treated with the proteasome inhibitor Bortezomib (BTZ), able to increase poly-ubiquitination, were used as an experimental control^52^ (Fig. 7a). Vehicle-treated G93A mice displayed abundant poly-ubiquitinated proteins whereas their levels were substantially reduced by compound **2a** (Fig. 7b). We next scored ubiquitin MFI levels in ventral horn MNs of G93A mice. MFI levels of ubiquitin fluorescence in vehicle-treated G93A MNs were higher than in compound **2a**-treated G93A mice, further confirming previous results^53^ (Fig. 7c and d). We next scored the Golgi apparatus in G93A mice by labeling lumbar sections for the cis-Golgi matrix protein GM130^54^. We observed that many MNs from vehicle-treated G93A mice displayed a reduction of GM130-covered areas, possibly indicating the organelle fragmentation^55^ (Fig. 7e). However, this phenomenon was substantially reverted when we scored MNs of compound **2a**-treated G93A mice (Fig. 7e).

Lysosomal alterations are early events in ALS^7,56^. We speculated that an impairment of the retromer should alter protease trafficking via CI-MPR, and therefore disrupt lysosomal homeostasis^57^. We determined whether a reduction of VPS35 levels in Neuro2a cells could affect lysosomal homeostasis, mimicking what was observed in ALS models^7,56^. We transfected Neuro2a cells with plasmids encoding the Sh56-RNAi to downregulate VPS35. After 24 h of starvation, we analyzed the lysosomes using Lyso-tracker^58^. We observed enlarged and abnormally distributed lysosomes in cells with VPS35 interference by Sh56 (Supplementary Fig. 12a–c). We next assessed that poly-ubiquitinated protein levels were doubled in Neuro2a cells receiving a VPS35-targeted RNAi, suggesting that loss of retromer functionality alters lysosomal homeostasis and the accumulation of poly-ubiquitinated proteins (Supplementary Fig. 12d, e)^21^. We asked whether the overexpression of SOD1^G93A^ would also result in similar lysosomal alterations by transfecting Neuro2a cells with plasmids encoding SOD1^G93A^, with or without compound **2a**. After 24 h of starvation, cells receiving only SOD1^G93A^ plasmids showed enlarged lysosomes, while as expected treatment with compound **2a** significantly reverted this phenotype (Supplementary Fig. 12f, g).

### Compound 2a counteracts SOD1G93A proteins aggregation

SOD1 mutations increase its propensity to aggregate in cells^59^. We checked whether compound **2a** could reduce the accumulation of SOD1^G93A^ aggregates in vivo. Starting at postnatal day 83, we injected G93A mice and WT littermates daily with compound **2a**. We scored motor performances in vehicle- and compound **2a**-treated G93A mice until day 103. As expected, mice receiving compound **2a** displayed a general attenuation of motor deficits (Supplementary Fig. 13a). We resolved SC protein extracts by blue-native polyacrylamide gel electrophoresis^60^, and probing them with anti-SOD1 antibodies we observed high molecular weight smears in G93A mice that were absent in WT litters. The intensity of such high MW entities was lowered in G93A mice treated with compound **2a** (Supplementary Fig. 13b, c). These results suggest that retromer stabilization mediated by compound **2a** increases lysosomal homeostasis in vitro and in vivo, decreases protein polyubiquitination and diminishes G93A aggregation.

### VPS35 expression in ASL SCs and in iPSCs-derived MNs from ALS fibroblasts

**Fig. 8:**
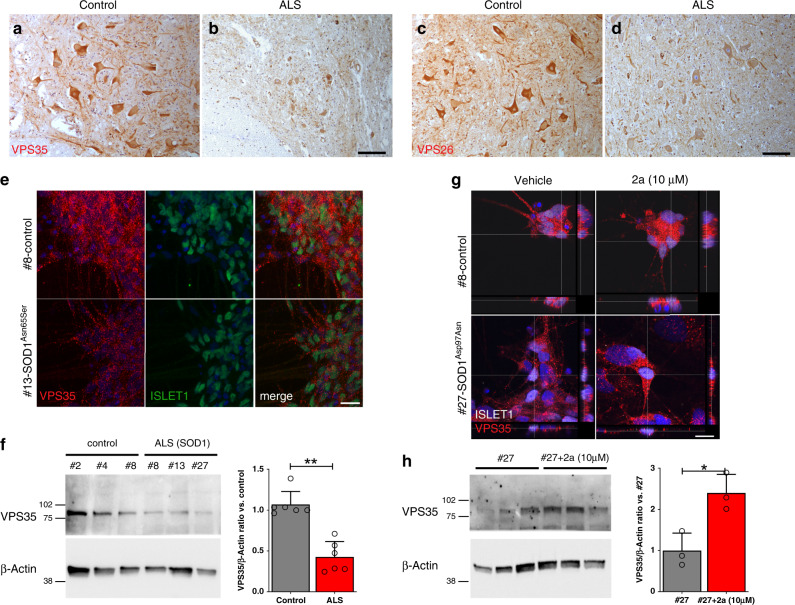
VPS35 levels in ALS iPSCs-derived MNs and postmortem biopsies. **a**, **b** IHC for VPS35 in alpha-MNs in representative sections from ventral horns of SCs biopsies from a non-neurological control (**a**) and an ALS patient (**b**). **c**, **d**, IHC for VPS26 in alpha-MNs sampled in adjacent sections from a non-neurological control (**c**) and an ALS patient (**d**). Analyses were done on four non-neurological control biopsies and five ALS biopsies examined over three independent experiments. **e** Maximum projections of representative confocal stacks (three independent experiments) for VPS35 and ISLET1 in representative cultures of iPSC-derived MNs from a healthy volunteer (#8) and an ALS patient (#13SOD1^Leu144Phe^). A representative WB for VPS35 and β-Actin in protein extracts from iPSCs-derived MN cultures (#8 SOD1^Asn65Ser^, #13SOD1^Leu144Phe^, #27 SOD1^Asp97Asn^ and age- and sex-matched healthy volunteers #2, #4, and #8) is shown in **f**. Quantifications derived from *n* = 3 controls (#2, #4, and #8) and from *n* = 3 ALS (#8, #13, and #27), means (±SD) are examined from two independent experiments (*p* = 0.005). **g** Representative cross-sectional analysis of confocal stacks (three independent experiments) for VPS35 and ISLET1 in cultured iPSCs-derived MNs from an healthy volunteer (#8) and from an ALS patient (#27 SOD1^Asp97Asn^) with or without compound **2a** (10 µM for 6 days). **h** Representative WB for VPS35 and β-Actin in iPSCs-derived MNs of an ALS patient (#27) treated with vehicle or with compound **2a** (10 µM for 6 days). Quantifications (means ± SD) derived from *n* = 3 wells/treatment and data are examined from one experiment (*p* = 0.017). Two-tailed Student’s *t*-test was used to determine the statistical significance in **f** and **h**. **p < 0.01. Scale bar: 150 µm in **d**, 25 µm in **e**, 15 µm in **g**.

We investigated VPS35 expression in human MNs located in the ventral horn of the SC (Fig. 8a). VPS35 immunoreactivity was substantially reduced in ALS MNs, confirming our observations in G93A mice (Fig. 8b). Parallel sections probed for VPS26 revealed a substantial reduction of VPS26 immunoreactivity in ALS samples (Fig. 8c, d).

We next generated iPSC lines from fibroblasts obtained from fALS patients carrying three *SOD1* gene variants (ALS 8: p.Asn65Ser; ALS 13: p.Leu144Phe; ALS 27: p.Asp97Asn), and from three healthy volunteers. Efficient reprogramming was assessed by labelling cultures for OCT3/4, SOX2, NANOG, TRA1-60, and SSEA4 (Supplementary Fig. 14). We observed a normal karyotype in ALS cell lines (Supplementary Fig. 15a). According to literature^61^ we induced the phenotype of caudal neuroepithelial OLIG2^+^PAX6^−^ progenitors in iPSCs (Supplementary Fig. 15b, c), and we differentiated them to acquire the OLIG2^-^ISLET1^+^ cell phenotype. We scored the ISLET1^+^ percentage among nuclei, observing similar rates of cell differentiation among controls and ALS cells (controls: 81.6% ± 9; ALS: 81.8% ± 12, Supplementary Fig. 15d, e). Using immunofluorescence and WBs, we observed that VPS35 levels in ISLET1^+^ MNs were reduced in ALS samples (Fig. 8e, f). We next selected a control cell line (#8) and an ALS cell line (ALS 27) for compound **2a** treatment. After establishing OLIG2^+^PAX6^−^ progenitors, we differentiated these cells in presence of compound 2a (10 µM, 6 days). Cells were labelled for ISLET1 and VPS35 and by confocal microscopy, and we scored VPS35^+^ puncta. ALS 27 cells displayed less VPS35^+^ puncta than controls, albeit both cell types significantly increased VPS35^+^ puncta when treated with compound **2a** (Fig. 8g). We next used WB for o confirm the ability of compound **2a** to increase VPS35 protein levels in ALS cells (Fig. 8h). Altogether, these results suggest that compound **2a** can be used to stabilize the retromer complex in ALS models.

## Discussion

We describe a substantial reduction of CRC proteins in MNs of G93A mice. Similarly, we observe a reduction of VPS35 levels in MNs scored in postmortem ALS patients, and in human iPSC-derived MNs from fALS patients. As retromer failures occur in AD^62^ and PD^17^ neurons, we speculate that the reduction of retromer levels observed in ALS represents one among many aberrant responses and deficits that affect neurons overwhelmed by misfolded/aggregated proteins.

This alteration occurs early in G93A mice, suggesting that the retromer could be an early therapeutic target in ALS, albeit future investigations are needed to identify mechanisms leading to retromer failure in ALS MNs.

The CRC core element of the retromer and the SNX dimer assemble to create a functional retromer complex^8,22,61^. Knockdown of a single CRC protein affects the expression of the other CRC members^15,21^. On the other hand, strengthening the interaction between proteins assembled in the CRC bolsters full retromer stability, and increases its functionality as elegantly described^20^.

Compounds binding to the interface between VPS35 and VPS29 and stabilizing the retromer assembly were identified by virtual screening. Among them, bis-isothiourea R55/**1** increased VPS35 levels (∼60%) in cells, and therefore the general stability of the retromer complex^20^. Treatment of neurons carrying pathogenic APP mutations with R55/1 reduced the accumulation of amyloid-β (Aβ) peptides as well as APP processing^20^.

We designed and synthesized a small array of phenyl bis-guanylhydrazone chaperones that interact with the CRC complex at the VPS35-VPS29 interface, as R55/**1**. We selected compound **2a**, due to its ability to increase VPS35 levels in Neuro2a cells and to cross the BBB. Through STD-NMR and WL-NMR experiments we experimentally proved the binding of **2a** to the VPS29-VPS35 heterodimer.

We assayed compound **2a** in vitro by overexpressing SOD1^G93A^ in cells. The overexpression of mutant SOD1 increases rates of cell death in MN-like NSC-34 cells^5^, and SOD1^G93A^ affects Neuro2a cell survival^63^. Compound **2a** significantly counteracted cell death and reduced LDH release in Neuro2a cells transfected with SOD1^G93A^ plasmids. Although we cannot rule out the possibility that compound **2a** modulates additional targets other than the CRC, we observed that it failed to rescue Neuro2a cell survival and to inhibit LDH release in presence of VPS35-targeted siRNA.

We used compound **2a** to restore retromer levels in G93A mice. Transgenic mice and their WT litters well tolerated intraperitoneally delivered compound **2a**, as we did not observe distress, aberrant behavior or pain in treated mice. This in vivo observation is coherent with in vitro low toxicity of compound **2a**, and with the absence of toxic effects in mice expressing AAV-VPS35^43^.

Neurons cannot divide, and therefore cannot dilute cytoplasmic misfolded/aggregated proteins that accumulate. Their removal is a complex task involving recognition proteins, chaperones, and eventually activation of degradation pathways such as the ubiquitin-proteasome system and the autophagy-lysosomal pathway^64^, both altered in ALS. Mutant SOD1 proteins associate with reduced expression of components of the ubiquitin-proteasome system^65^. C9orf72, an ALS causative gene, is a regulator of autophagy^66^ and a keeper of lysosome homeostasis^67^. Lysosomal deficits are well documented in G93A mice and starting from day 40, the levels of CTSD are significantly reduced in MNs^7^ from these mice. The maturation of this lysosomal enzyme involves CI-MPR and a functional retromer^21^. We observed increased levels of both CI-MPR and CTSD in MNs of G93A mice treated with compound **2a**. Therefore, we hypothesized that increasing retromer stabilization could increase the delivery of CTSD to the lysosomal compartment and finally the lysosomal functionality. These data are far from conclusive, and further experiments aimed to inactivate CTSD in G93A mice are needed to investigate its contribution to the degradation of misfolded proteins.

Golgi fragmentation is commonly observed in MNs of sALS and fALS patients^35,68^. Using compound **2a** we observed a substantial attenuation of Golgi fragmentation in SOD1^G93A^ overexpressing cells and in G93A mice. The mechanism(s) that connect retromer stabilization to the rescue of Golgi fragmentation are unknown, although experimental evidence suggests a functional link between a protein belonging to Vesicle Associated Membrane Protein (VAMP)-associated proteins (VAP) VPSs and Golgi homeostasis. A mutant form of *VAP-B* (P56S) was described in a family of ALS patients^69^, and VAP-B levels are dampened in MNs of G93A mice^70^. The retromer complex interacts with VAP-B and such interaction occurs in regions in which the ER makes contacts with endosomes^71^. It is tempting to speculate that increasing the retromer stability with compound **2a**, we can also restore VAP-B functionality, indirectly restoring homeostasis. Further investigations will help to dissect the interaction between VAPs and retromer in ALS mice.

Compound **2a** attenuated locomotion impairment in G93A mice, and significantly increased the number of surviving MNs. These functional effects correlate with the ability of **2a** to increase CRC protein levels in MNs, but further experiments with VPS35 conditional KO mice will further validate in vivo the mechanism of action of compound 2a.

Lysosomal alterations probably concur to increase the accumulation of poly-ubiquitinated proteins in the lumbar SC^72^. Such pathological phenomenon was significantly reduced in vitro and in vivo by compound **2a**. We do not know whether retromer failure is the leading trigger inducing the loss of lysosomal functionality in ALS, but we observed that either VPS35 knockdown or overexpression of SOD1^G93A^ in Neuro2a cells increased poly-ubiquitinated protein levels. We acknowledge that failure of the retromer complex could lead to lysosomal deficits, autophagy alteration and exaggerated protein poly-ubiquitination by synergizing with other detrimental pathways operating in the ALS pathophysiology. Similarly, G93A mice receiving compound **2a**-driven retromer stabilization showed a substantial reduction of high molecular weight SOD1 aggregates.

Therefore, pharmacological retromer stabilizers could be useful to delay degenerative processes occurring in ALS.

## Methods

### Docking experiments

The docking analysis was performed with AutoDock4.2 software^73^ building the coordinates of the VPS29-VPS35 heterodimer^74^ (pdb-id 2r17). Each ligand was drawn with the program “Marvin Sketch v20.13.0” (Chemaxon packages; https://chemaxon.com/), converted in a 3D structure and then transformed in the *pdbqt* format with the addition of partial charges and rotatable bonds (http://mgltools.scripps.edu). A cubic docking grid centered around the side chain of Gln530 of VPS35 with 15 Å side length was used for explorations. We performed 40 independent, global-local Lamarkian genetic-algorithm runs for each tested compound, extracting the conformation, the binding energy and the calculated Kd of the best pose together with the number of poses clustered around such best conformation (Python Molecular Viewer http://mgltools.scripps.edu). Additionally, we utilized a sigmoidal rectification function shown in Supplementary Fig. 4a to weight the calculated Kd with recurrence of best docking poses and generate a normalized Kd, as explained in the main text.

### Druggability parameters

pKa and clogP values were calculated using the “Calculator Plugins” module of the ChemAxon's Marvin suite (https://chemaxon.com/marvin-archive). We used the default parameters without any correction.

### Synthesis

*General procedures*: ^1^H-NMR spectra were recorded on a Bruker Avance 400 MHz instrument in CDCl_3_, CD_3_OD, or D_2_O as solvent at 400 MHz. ^13^C-NMR spectra were recorded in CDCl_3_, CD_3_OD, or D_2_O as solvent at 101 MHz. Coupling constants are expressed in Hertz and are rounded to the nearest 0.1 Hz. LC-MS data were collected with a Waters Acquity^TM^ Ultra performance LC equipped with an Acquity UPLC^TM^ HSS T3 column (2.1 × 50 mm, 1.8 µm) and a SQD detector. Purifications were carried out either by flash chromatography on silica gel (particle size 60 μm, 230–400 mesh), on Kieselgel, or by Biotage^TM^ flash chromatography [Biotage columns Si-25-M (150 × 25 mm; silica gel (40–63 μm), flow rate 25 mL/min)], or by Biotage^TM^ C_18_ reverse phase chromatography [Biotage column C_18_HS (150 × 25 mm; KP-C_18_-HS (35–70 μm), flow rate 25 mL/min)]. Some final compounds were purified by C_18_ reverse phase semi-preparative HPLC using a Waters X-Bridge column (19 mm × 15.0 cm, 5 μm). Melting points were determined with a Stuart Scientific SMP3 melting point apparatus. Solvents were distilled and dried according to standard procedures, and reactions requiring anhydrous conditions were performed under nitrogen or argon atmosphere.

### Synthesis of bis-1,3-phenyl guanylhydrazone **2a**

Compound **2a** (280 mg, 0.88 mmoles, 88% yield, ≥95% purity, m.p. 200–202 °C, white solid) was prepared in a single step from commercially available isophthalic aldehyde and aminoguanidine dihydrochloride. Diode Array LC trace, TIC trace and ES scan for compound 2a are available in Supplementary Fig. 4e; ^1^H-NMR and ^13^C-NMR are provided in the Supplementary Fig. 4f and g, respectively.

### Animals and treatments

Mice were maintained under pathogen-free conditions at San Raffaele Hospital mouse facility (Milan, Italy). All efforts were made to minimize animal suffering and to reduce the number of mice used in accordance with the European Communities Council Directive of 24 November 1986 (86/609/EEC). The Ethics Review Committee approved experimental protocols according guidelines from the Italian Ministry of Health and from the Institutional Animal Care and Use Committee of the San Raffaele Scientific Institute (protocol number 704/2015PR). Transgenic mutant SOD1 mice carrying the SOD1^G93A^ allele (strain B6SJL-TgN[SOD1G93A]1GUR)^75^ were purchased from Jackson laboratories, while C57BL/6J mice, used for breeding and experimental purposes, were purchased from Charles River (Italy). Both transgenic and WT mice had food and water freely available in the home-cage. The holding room was on a 12-h light–dark cycle, the temperature of the room was 22 ± 0.2 °C. Starting from day 30 we intraperitoneally injected compound 2a (10 mg/Kg) or vehicle in G93A mice as well as in C57BL/6J mice for 70 days. Additional experiments were performed injecting mice from day 83 to day 103. An operator blind to the treatment performed injections and manipulation of mice. At the day of the sacrifice, mice received an overdose of anesthetic drugs and they were perfused via vascular system with saline followed by 70–80 ml 4% paraformaldehyde (PF) in PBS, pH 7.2 (Sigma–Aldrich). Spinal cords were coronally sliced at ~6-mm thickness and postfixed in 4% PF in PBS, pH 7.2 for 12 h at +4 °C. Tissues were cryoprotected in PBS 1X/30% Sucrose (Sigma–Aldrich), embedded in OCT inclusion media and stored at −80 °C before processing. Lumbar spinal cords were 12-µm sectioned, labelled and digital images were acquired every 330 µm in a region encompassing 2.4 mm of the lumbar spinal cord. We determined numbers of mice by preliminary results or literature precedent^76^.

### Motor function

Motor activity of mice receiving compound **2a** or vehicle was assessed on 50-, 60-, 70-, 80-, 90-, and 100-days-old animals. Briefly, mice were trained 1 min on a static rotor and 1 min at constant speed (4 rpm) for two times and then we ran two trials performed over two consecutive days (one per day). Each trial consisted of 6-test sessions with 15 min interval between sessions. For each session, we placed mice on an accelerating rotor (4–40 rpm) and the latency to fall was recorded, with a maximum limit for individual animal set at 600 seconds.

### Mass spectrometry quantitative analysis of compound 2a

Quantification of compound **2a** in CNS extracts of C57BL/6J mice was performed at ProMeFa facility (Proteomics and Metabolomics Facility) of San Raffaele Scientific Institute. Wild-type C57BL/6J males mice (P60) were intraperitoneally injected with compound **2a** (10 mg/kg) or vehicle, daily. We sacrificed mice at the following time points: 1, 7, 15, and 30 days. Before brains collection, mice received saline perfusion. Brains were weighted (~350 mg each) and rapidly frozen at −80 °C. The extraction was performed with methanol and each biological sample was resuspended in 60 µl of LC/MS grade water and 5 µL were analyzed by LC-MS/MS using the UPLC 1290 (Agilent Technologies) coupled to the TripleTOF 5600+ mass spectrometer (SCIEX). The mass spectrometry analysis was carried out in positive mode, in the range of 50-500 m/z for both the TOF-MS scan and the product ion scan, with a selected product ion of 247.1. A Waters ACQUITY UPLC BEH HILIC column (2.1 × 10mm, 1.7 µm) was used for the chromatographic separation through a gradient of solvent A (acetonitrile + 0.1% formic acid) and solvent B (water + 0.1% formic acid) from 2% up to 29% B in 5 minutes with a flow rate of 600 µl/min. Calibration curve in brain was constructed by plotting the peak area versus corresponding quantities of compound 2a (0; 0.1; 0.26; 1 nmol, run in quadruplicates, R2 = 0.9443), using MultiQuant 2.1 software (SCIEX).

### NMR spectroscopy

NMR spectra were acquired at 298K on a Bruker Avance NEO 700 MHz spectrometer equipped with a Z-gradient cryoprobe. The spectra were processed with the Bruker TOPSPIN 4.0.5 software packages. For NMR studies of compound **2a** with VPS29-VPS35, 5 μl from a DMSO-d_6_ stock 20 mM solution were dissolved in 175 μl of buffer (50 mM KH_2_PO_4_, 50 mM Na_2_HPO_4_, pH 8.0, 150 mM NaCl, 2 mM DTT) and 20 μL of ^2^H_2_O. Saturation Transfer Difference spectra (^1^H spectral window = 16 ppm; relaxation delay = 3.0 s; number of points = 32K) were acquired with 1024 scans with on-resonance irradiation at −1.0 ppm for selective saturation of protein resonances, and off-resonance irradiation at 40 ppm for reference spectra. A train of 40 Gaussian shaped pulses of 50 ms with 1 ms delay between pulses were used, for a total saturation time of 2 s. 1D ^1^H spectra were recorded using a 1 s presaturation pulse with a B_1_ power of 200 Hz. WaterLOGSY experiments (WL) were acquired using the ePHOGSY sequence^33^. WL experiments (^1^H spectral window = 16 ppm; relaxation delay = 3.0 s; number of points = 16K, scan = 1024) employed a 20 ms selective Gaussian 180° pulse at the water signal frequency and a NOE mixing time of 1 s. Both for STD and WaterLOGSY, FIDs were multiplied by an exponential weighting (lb = 5 Hz) before Fourier transformation.

### Cell lines, culture conditions, and treatments

Neuro2a cells (ATCC^®^ CCL-131™) were seeded in Dulbecco’s modified Eagle’s media (DMEM, Gibco) supplemented with FBS 10% (Gibco), L-glutamine (Gibco) 2 mM and penicillin/streptomycin 1% (Gibco). Depending on the experimental setting, we used 6-well, 12-well or 96-well dishes. Neuro2a were grow at 37 °C in a humidified 5% CO_2_ atmosphere. All experiments were carried out at least in triplicate. Compounds and transfections were done on cells showing 60–70% of confluence. Compounds were usually kept on cells for 48 h; Bortezomib (BTZ) was used at 10 nM for 6 h to inhibit proteasome. Transient transfections were obtained using lipofectamin LTX reagent (Thermofisher) with the following plasmids: pCMV-LacZ; pcDNA3.1(+)SOD1G93A;^77^ pcCDNA3.1(+)VPS35; pCAAG-GFP according the manufacturer’s instructions.

VPS35 short interference: we used Lipofectamine LTX (Thermofisher) to transfect Neuro2a cells with the following plasmids: plKO short interference scramble; TRCN0000111556 (Sh56); TRCN0000111558 (Sh58); TRCN0000111559 (Sh59), (Mission, Sigma–Aldrich). Protein and total mRNA extracts were obtained according the experimental design.

Cell survival experiments were run on 96-well plates using Cell Counting Kit-8 (CCK8) survival kit (96992-Sigma–Aldrich) according the manufacturer’s recommendations. Compound **2a** cytotoxicity was tested on Neuro2a cells receiving increasing amounts of compound **2a** (1, 10, 50, 100, 200, 500, and 1000 µM) for 48 h. Data were acquired using an Epoch spectrophotometer equipped with the GEN5 (2.03.1) software (Agilent).

LDH cytotoxicity was run on 96-well plates using LDH-Glo™ Cytotoxicity Assay (Promega). Briefly, Neuro2a cells were transfected with plasmids encoding pCMV-LacZ; pcDNA3.1(+)SOD1G93A^77^ with or without VPS35 short interfering plasmids (Sh56) or compound **2a** (10 µM). After 48 h, 5 µl of supernatants were 100-fold diluted in LDH storage buffer (Tris-HCl 200mM, 10% Glycerol, 1% BSA, Sigma–Aldrich) and used to determine LDH, according to manufacturer’s recommendations. Maximum LDH Release Control was obtained lysing untreated Neuro2a cells with 2 μL of 10% Triton X-100 (Sigma–Aldrich). In each experiment we calculated$\% \,{\mathrm{cytotoxicity}} = \frac{{{\mathrm{Exp}}.{\mathrm{LDH}}\,{\mathrm{release}} - {\mathrm{medium}}\,{\mathrm{background}}}}{{{\mathrm{Max}}\,{\mathrm{LDH}}\,{\mathrm{release}}\,{\mathrm{control}} - {\mathrm{medium}}\,{\mathrm{background}}}}$, and we run parallel positive controls treating cells with 200 µM H_2_O_2_. Data were acquired using a Victor3 spectrophotometer equipped with Wallac 1420 software (PerkinElmer)

Cycloheximide (CHX) assay was done on Neuro2a cells seeded in 6-well plates and incubated with compound **2a** (10 µM) or vehicle for 24 h. We next added CHX (Sigma–Aldrich) at 10 µg/mL and cells were collected at 0, 2, 4, and 8 h for the time-course assay of VPS35 levels by WB.

### Generation of human iPSC lines and derived MNs

Skin biopsies from ALS patients and healthy volunteers were performed under local anesthesia, after informed consent at San Raffaele Hospital (BANCA INSPE/8-10-19 and MND Genotipo Fenotipo/16-5-19, approved by the Ospedale San Raffaele Etic Committee). Primary fibroblasts were grown in Dulbecco’s modified Eagle’s medium with Glutamax I (GIBCO). Human iPS cell lines were generated using non integrating Sendai virus, maintained in feeder-free conditions in mTeSR-1 (Stem Cell Technologies) on embryonic stem cell (HESC) Matrigel (Corning). MNs were generated following the protocol published by Du et al.^78^ with minimal modification. Briefly, when hiPSCs reached confluence, they have been detached using EDTA and plated 1:4 on Matrigel ES coated dishes in mTeSR-1 supplemented with ROCK inhibitor (StemMACS, Miltenyi Biotec). On the following day the medium was replaced with a chemically defined neural medium: DMEM/F12, Neurobasal medium at 1:1 ratio, 1% B27, 0.5% N2 (all from Gibco, ThermoFisher Scientific), 1% P/S (Gibco), 1% Glutamax (Gibco) and 0.1 mM Ascorbic Acid (Sigma–Aldrich). CHIR99021 (3 μM, Tocris), DMH1 (2 μM, Tocris) and SB431542 (2 μM, Miltenyi Biotec) were added to the medium. The culture medium was changed every other day, until day 6. On day 7, cells have been dissociated with Dispase (1U/mL), split 1:4 and kept on Matrigel growth factor reduced (MaGR) for 6 days in the basal neural medium described above, supplemented with 1 μM CHIR99021, 2 μM DMH1, 2 μM SB431542, 0.1 μM Retinoic Acid (RA, Sigma–Aldrich) and 0.5 μM Smoothened Agonist, (SAG, Calbiochem). On day 13, cells have been dissociated with Dispase (1U/mL), split 1:4, and kept for 6 days in neural medium supplemented with 3 μM CHIR99021, 2 μM DMH1, 2 μM SB431542, 0.1 μM RA, 0.5 μM SAG, and 0.5 mM Valproic Acid (VPA, Tocris). To induce MN differentiation, cells were dissociated with Dispase (1U/mL) and cultured in suspension for 6 days in neural medium with 0.5 μM RA and 0.1 μM SAG. On day 25, cells were dissociated into single cell with Accumax (Sigma–Aldrich) and plated on Matrigel Growth Factor Reduced (Thermofisher) coated plates, in neural medium with 0.5 μM RA, 0.1 μM SAG, 0.1 μM Compound E (Calbiochem) until day 35. Half of the medium has usually been changed every other day. The karyotyping analysis was performed by ISENET Biobanking service unit in Milan, Italy (www.isenetbiobanking.com).

### Immunoblotting

Lumbar SCs were dissected after mice received saline perfusion and were rapidly homogenized in the following lysis buffer: Tris-HCl 10 mM pH 8, EDTA pH 8 1 mM, NaCl 100 mM, NP40 1% and protease inhibitor cocktail (Sigma–Aldrich). Protein extracts were obtained using a tight-fitting glass Potter tissue grinder (1 mL; Wheaton) and then sonicated at a frequency of 20 kHz (10 times-1s). Protein lysates from cells cultures were collected from plates receiving PBS 1× washing and subsequently the lysis buffer followed by sonication. Protein concentrations were measured by BCA protein assay kit (Thermofisher) according to the manufacturer’s recommendations. Ten µg of protein extract was loaded on 10% Mini-PROTEAN TGX Stain-Free precast gels for PAGE (Bio-Rad). Gel electrophoresis was performed at 100 V for 2 h. Gels were transferred on Immobilon®-P PVDF membrane according to the manufacturer’s instructions (Millipore). Blots were blocked in 5% BSA in Tris buffered saline plus 0.1% Tween-20 (TBS-T) for 1 h before receiving primary antibodies: goat α-VPS35 1:800 (ab10099, Abcam); rabbit α-VPS26 1:1000 (ab23892 Abcam), rabbit α-VPS26b 1:1000 (15915-1-AP, ProteinTech); rabbit α-VPS29 1:1000 (ab236796 Abcam); mouse α-βActin 1:25000 (Sigma–Aldrich); mouse α-Golgin97 1:1000 (Invitrogen); mouse α-Ubiquitin 1:500 (Merk); rabbit α-CD14 1:1000 (Bioss Inc); mouse α-Tubulin 1:5000 (Immunological Sciences MAB-80143) overnight at 4 °C on shaker. Following several washes with TBS-T, blots were incubated in appropriate HRP-labeled secondary antibodies (Bio-Rad) for 1 h at room temperature. Visualization of protein bands was obtained using ClarityMax-ECL (Bio-Rad) according to the manufacturer’s instructions and images were acquired on ChemiDoc (Bio-Rad). Quantifications were done using the Image Lab 6.0.1 software (Bio-Rad).

### Blue-Native gel electrophoresis

G93A mice and their WT litters were injected daily with vehicle or compound **2a** (10 mg/kg, from day 83 to day 103). Lumbar spinal cords dissected from saline-perfused mice were lysed in Tris-HCl 10 mM pH 8, EDTA 1 mM pH 8, NaCl 100 mM, NP40 1% and protease inhibitor cocktail (Sigma–Aldrich). Extracts were obtained using a tight-fitting glass Potter tissue grinder (1 mL; Wheaton). We incubated 250 µL of each extract with 100 mM Iodoacetamide (Sigma–Aldrich). Samples were mixed with 4× Sample NativePAGE (Thermofisher) sample buffer and the G-250 additive (Thermofisher) and proteins were resolved on native 4–15% Bis-Tris gel (Bio-Rad) with Dark Blue Cathode buffer (Thermofisher) at 150 V for 1/3 of gel length. We replaced the running buffer with Light Blue Cathode Buffer to complete the run. At the end of the electrophoresis, gels were washed with 5 mM tris(2-carboxyethyl)phosphine (Sigma–Aldrich) for 15 min and then proteins were transferred to nitrocellulose and fixed on the membrane by 8% acetic acid for 15 min. Rabbit α-SOD1 (1:2000, Genetex) was incubated overnight as above described. Visualization of proteins was performed using ClarityMax-ECL (Bio-Rad) according to the manufacturer’s instructions and images were acquired on ChemiDoc (Bio-Rad).

### Immunohistochemistry, immunofluorescence, and morphometric analyses

Paraffin embedded SCs were obtained from ALS patients and non-neurological controls from the Target ALS Human Postmortem Tissue Core. Sections were stored at the INSPE tissue Bank (San Raffaele Scientific Institute). Sections were incubated with a polyclonal VPS35 antibody (1:400 Abcam ab97545) and with the polyclonal VPS26 antibody (1:200, Abcam ab23892) after rehydration and antigen retrieval (slides were cooled at 97 °C in TRIS EDTA pH = 9 by water bath). Peroxidase-diaminobenzidine (DAB) reaction was used for detecting primary antibody, biotinylated anti-rabbit IgG (H+L) (1:500 Vector laboratories) and avidin-biotin complex (ABC 1:100 Vector laboratories). An operator that was blind to the disease status and demographic data subsequently evaluated these sections.

Sections from mice or glass covers containing cells were washed three times in PBS 1× (Lonza, 5 min each), and incubated in the following blocking mix: PBS 1×/FBS 10% (Invitrogen), BSA 1 mg/mL (Sigma–Aldrich), TritonX100 0.1% (Sigma Aldrich), for 1 h at room temperature. Antibodies were diluted in blocking mix and incubated at +4 °C overnight according manufacturer’s instructions. The following day, sections were rinsed in PBS and fluorescent secondary antibodies—i.e. never deriving from the species from which the primary antibodies are derived- (Alexafluor conjugated) diluted in blocking mix, were used according to the manufacturer’s instructions. Slides were incubated in Hoechst 33342 (Sigma–Aldrich) for nuclei counterstaining. When necessary, we performed antigens retrieval on tissue sections by boiling samples in 10 mM sodium citrate (pH 6) for 5 min. The following antibodies and working concentrations were used: rabbit α-Iba1 1:500 (Wako); rabbit α-VPS35 1:1000 (ab97545 Abcam); goat α-VPS35 1:800 (ab10099 Abcam); rabbit α-VPS26 1:1000 (ab23892 Abcam); rabbit α-Sortilin 1:1000 (ab16640 Abcam); mouse α-CI-MPR 1:100 (Novus bio. NB-300-514), mouse α-NeuN 1:800 (Millipore MAB377); mouse α-Ubiquitin 1:500 (Millipore MAB1510); mouse α-Golgin97 1:700 (Thermofisher A21270); rabbit α-ISLET1:1000 (ab20670 Abcam); goat α-ChAT 1:200 (Millipore ab144p); chicken α-GFP 1:1000 (Invitrogen); α-SOX2 1:100 (R&D, MAB2018); goat α-NANOG 1:200 (R&D, AF1997); mouse α-OCT3-4 1:500 (Santa Cruz, sc-5279); mouse α-SSEA4 1:400 (Millipore MAB4304); mouse α-TRA1-60 1:200 (Millpore MAB4360); chicken α-Neurofilament-medium 1:500 (Biolegend PCK-593P); rat α-MBP 1:100 (kindly provided by Dr. A. Bolino); mouse α-GM130 1:100 (BD biosciences, 610823); rat α-CTSD 1:100 (R&D system MAB1029). Tyramide Signal Amplification (TSA, PerkinElmer) was used, when appropriate, to improve fluorescent intensity in single and double immunofluorescence.

Quantification of nerves degeneration was done on sciatic nerves obtained from saline-perfused mice. Tissues were fixed in 2% buffered glutaraldehyde (Sigma–Aldrich), postfixed in 1% osmium tetroxide and cut to obtain 1 µm thick sections. We stained sections with toluidine blue and specimens were blindly examined by light microscopy using ×40 objective. For each nerve, we merged images to obtain the entire nerve cross section. Five consecutive nonoverlapping semi-thin sections from each sample were analyzed with NHI-Image J software (US National Institutes of Health). Degenerating fibers were counted in the entire nerve cross section.

Quantification of myelin was done on sagittal sciatic nerves by Luxol fast blue (LFB). Briefly, nerve sections were incubated the ethanol series before receiving 0.1% LFB overnight at 60 °C. Sections were then incubated 5 min in 0.05% lithium carbonate before receiving the ethanol series and xylene. Bright field microcopy of nerves at ×63 was used for quantification. Positive myelin staining is expressed as a percentage of the total area examined.

### Imaging

We used Olympus, BX51 with the following objectives: ×20, ×40, and ×63 equipped with the following cameras: Leica CCD Microscope DFC3000 G and DMC2900 to obtain 16-bit light and fluorescence images (1296x966 pixels). Fluorescence images were merged using Photoshop (Adobe) CS4 or NHI-Image J software (US National Institutes of Health). We obtained confocal images using Leica SP8 equipped with ×40 and ×64 objectives and super-sensitive HyD detectors. We recorded each fluorescence signal as square 8-bit images (1024 × 1024 pixels). Images were postprocessed to generate maximal projections of Z-stacks (acquired with a 0.4–0.8 µm step) and cross-sectional profiles of the Z-stack (acquired with a 0.3 µm step) and pseudo-colored using Leica Application Suite X (2.0.0.14332).

### Quantification of MFI in tissue MNs

Fluorescence intensity levels of VPS35, VPS26, CI-MPR, Sortilin, CTSD and Ubiquitin were calculated using NHI-Image J software (US National Institutes of Health) according to the following protocol: stacks of confocal images were used to generate the maximal projections using the Leica Application Suite X (2.0.0.14332) software and then postprocessed by NHI-Image J to crop single NeuN^+^ MNs from the ventral horn of the spinal cord. We next performed background (calculated on adjacent slices only receiving secondary antibodies) subtraction for each channel, before applying the NHI-Image J mean filter (with radius 2.0 pixels) to calculate mean NeuN fluorescence levels in individual MNs. Using this approach, we established a region of interest (ROI) to gate single MNs. On this ROI, we measured mean fluoresce levels (MFI) of target proteins.

### Morphological assessment of Golgi apparatus in Neuro2a

Golgi morphology was assessed in control or in Neuro2a cells receiving G93A plasmids with or without compound **2a** (10 µM) as well as in GFP-transfected cells as additional control. We used Golgin97 immunofluorescence to label trans-Golgi network membranes. We classified Golgi morphology as follow: normal (polarized network), intermediate (partially fragmented) or fragmented (severely fragmented with Golgi stacks dispersed in the cytoplasm throughout). In each experiment, cells were randomly sampled across three coverslips. Golgi subclasses were expressed as a percent of the total number of Golgi scored for each experimental condition.

### Micro electrode array (MEA) recordings

Primary neuronal cultures were established from the cerebral cortex of E16.5 C57BL6J embryos. Upon dissection, cells were plated on MEA biochips (60 electrode MEA biochips with 200 µm electrode spacing and 30 µm electrodes diameter with an integrated reference electrode, Multichannel Systems GmbH) at the density of 3 × 10^5^ cells/MEA and kept in Neurobasal medium supplemented with B27 for 14 days. At day 14 neuronal cultures were exposed to increasing concentration of compound **2a** (1, 3, 10, 30, and 100 µM) in Krebs–Ringer–Hepes buffer (KRH, 130 mM NaCl, 5 mM KCl, 1.2 mM KH_2_PO_4_, 1.2 mM MgSO_4_, 2 mM CaCl_2_, 25 mM Hepes, 0.6% glucose, 1% glutamine, Sigma–Aldrich). Firing activity was recorded using a pre-amplifier stage (MEA-1060-Inv-BC-Standard, gain: 55, bandwidth: 300 Hz–8 kHz, MCS GmbH), an amplification and filtering stage (FA64S, gain 20, bandwidth: 10 Hz–8 kHz, MCS GmbH) and a data acquisition device (sampling frequency: 25 kHz). Then, off-line signal processing was performed, and raw data were analyzed by MC-Rack Software version 4.6.2 (MCS GmbH).

### Real-time PCR

Lumbar spinal tracts dissected from saline-perfused mice or Neuro2A cells were lysed to extract total RNAs using the RNeasy Mini Kit (Qiagen) according to the manufacturer’s recommendations, including DNase (Promega) digestion. We synthetized the cDNA using ThermoScript RT-PCR System (Invitrogen) and Random Hexamer (Invitrogen), according to the manufacturer’s instructions. The LightCycler 480 System (Roche) and LightCycler 480 SYBR Green I Master Mix (Roche) were used for real-time PCR experiments. Normalization was obtained using the housekeeping gene Histone H3:

*F: 5*′*-GGTGAAGAAACCTCATCGTTACAGGCCTGGTAC-3*′

*R: 5*′*-CTGCAAAGCACCAATAGCTGCACTCTGGAAGC-3*′

The following specific primers were used for gene expression analysis: VPS35F: *5*′*-GTGCCGTGGTGTGCAGCATCCG-3*′VPS35R: *5*′*-ATGCTGCATCCGCACCCAGAGC-3*′VPS26A F: *5*′*-GCTAAGTATGAAATAATGGATGGGGC-3*′VPS26A R: *5*′*-CTTGTTCACATCTCTCATCGTGGGG-3*′VPS29F: *5*′*-CCAGCACATCCTCTGCACCGGC-3*′VPS29R: *5*′*-CCACGGAATAACTTGGTGTCCGTG-3*′.

### Statistics

We express data as the mean value (±standard error of the mean (SEM) or ±standard deviation (SD)) of independent experiments. We assessed normality of data applying either Kolmogorov–Smirnov test (with Dallas–Wilkinson–Lille for *P*-value) or D'Agostino & Pearson omnibus normality test. Comparisons were made using the following tests: unpaired *t*-test, one-way or two-way analysis of variance (ANOVA) tests, followed by Tukey's multiple Comparison test or by Bonferroni multiple comparison test. Statistical analyses were performed using PRISM5.01, PRISM8.4.2 (GraphPad Software, La Jolla, CA, USA) or BioVinci 1.1.5 (BioTuring Inc.). Values lower than 0.05 were considered statistically significant.

### Reporting summary

Further information on research design is available in the Nature Research Reporting Summary linked to this article.

## Supplementary information

Supplementary Information

Reporting Summary

## Data Availability

Authors can confirm that all relevant data are included in the paper and its supplementary information. Source data are provided with this paper. The data that support the findings of this study are available from the corresponding author (luca.muzio@hsr.it), upon reasonable request. Source data from all representative blots shown in this study as well as data underlying all quantitative analysis performed in this study are provided with the manuscript as a Source Data file. Source data are provided with this paper.

## Source data

Source Data
