# Supplementary material for: Retromer stabilization results in neuroprotection in a model of Amyotrophic Lateral Sclerosis
Source: Nat Commun. 2020 Jul 31;11:3848. doi: 10.1038/s41467-020-17524-7 (PMC7395176; doi:10.1038/s41467-020-17524-7)
Supplement: Supplementary file 1 — Supplementary Information [file 41467_2020_17524_MOESM1_ESM.pdf]

Supplementary Information

Retromer stabilization results in neuroprotection in a model of Amyotrophic Lateral Sclerosis

Luca Muzio, Riccardo Sirtori, Davide Gornati, Simona Eleuteri, Andrea Fossaghi, Diego Brancaccio, Leonardo Manzoni, Linda Ottoboni, Luca De Feo, Angelo Quattrini, Eloise Mastrangelo, Luca Sorrentino, Emanuele Scalone, Giancarlo Comi, Luciana Marinelli, Nilo Riva, Mario Milani, Pierfausto Seneci, Gianvito Martino.

Correspondence to: muzio.luca@hsr.it

This PDF file includes:

Supplementary figures 1 to 15

Supplementary Figure 1

VPS35/26 distribution in ventral horn MNs of G93A mice

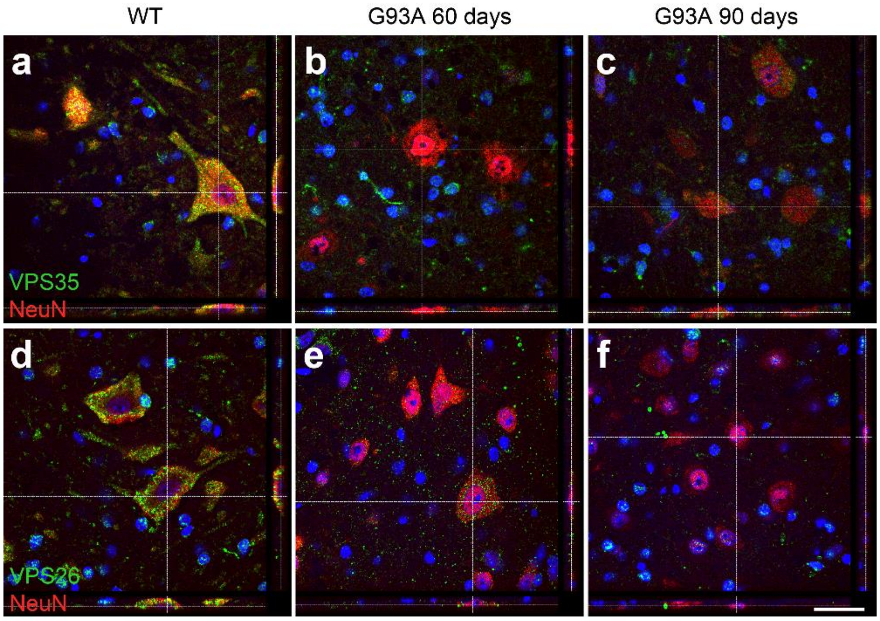

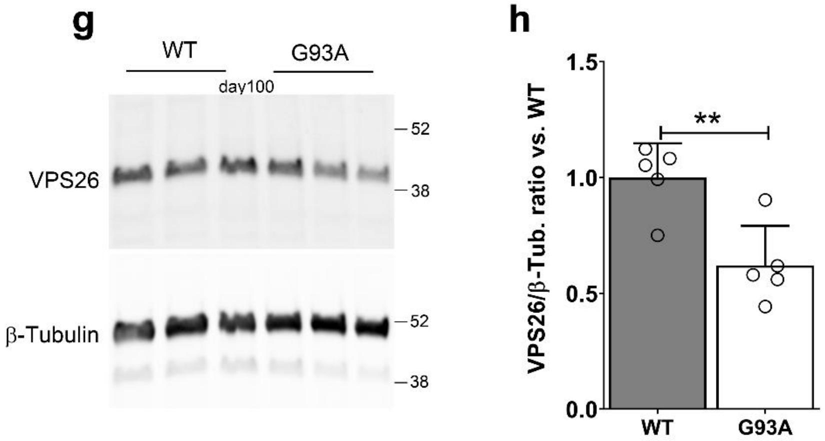

Representative cross sections of confocal stacks acquired with a step of 0.3 µm from WT (a, d) and G93A (b, c, e and f) sampled at 60 and 90 days, respectively. a-c show sections labelled for VPS35 and NeuN (n=3 independent mice/group). d-f show sections labelled for VPS26 and NeuN (n=3 independent mice/group). Imaging was done in the ventral horn of the lumbar SC; orthogonal views show the distribution of VPSs^{+} in single NeuN^{+} MNs (data were examined over 3 independent experiments). Panel g shows a representative WB of lumbar SC protein extracts from WT and G93A mice sampled at day 100 and probed with anti-VPS26 antibody and β-Tubulin; h, quantifications (means ± SD) of normalized VPS26 levels (ratio versus WT, n=5 independent mice/group from 2 independent experiments, p=0.0056). Two-tailed Student's test was used to determine the statistical significance of data, ** p<0.01. Scale bar 20 µm.

Supplementary Figure 2

VPS35/26 in the motor cortex of G93A mice

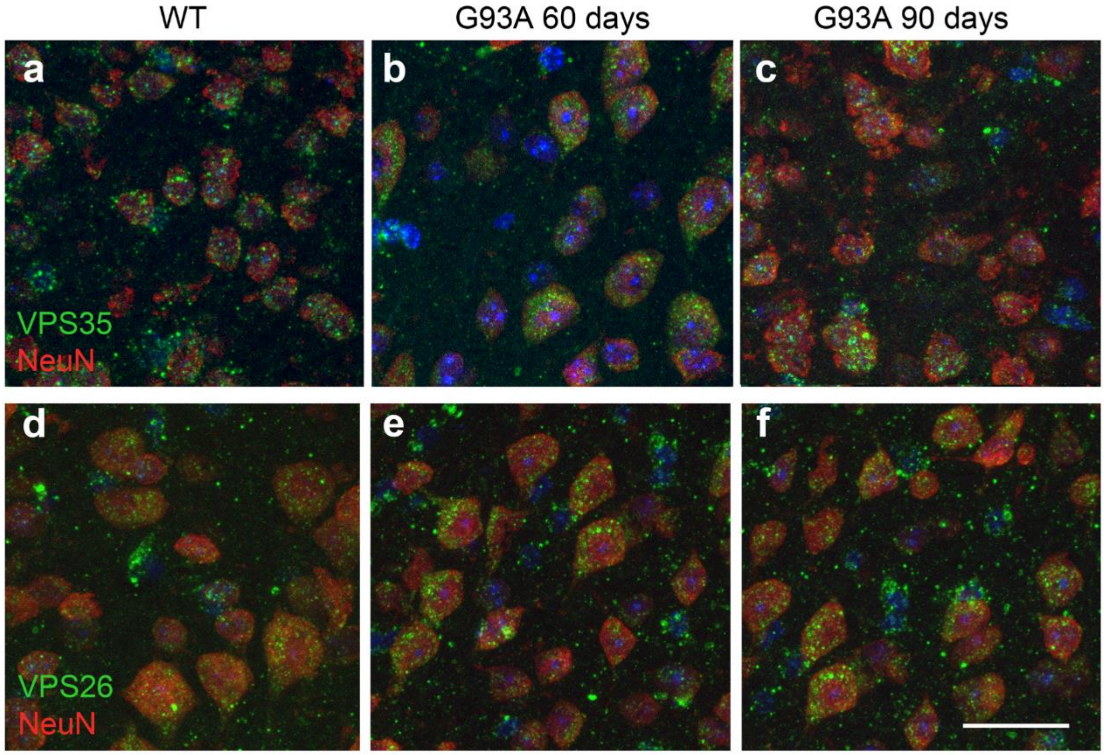

a-c, representative maximal projections of confocal stacks from WT (a, d) and G93A (b, c, e and f, 60 and 90 days). Imaging was done in the motor cerebral cortex labelled for VPS35 and NeuN, (n=3 independent mice/group, a-c). Adjacent sections (d-f) were labelled for VPS26 and NeuN and images are shown in panels d-f, (n=3 independent mice/group). Data are representative of 2 independent experiments. Scale bar 60 µm.

Supplementary Figure 3

pKa ranges for isothioureas and guanylhydrazones

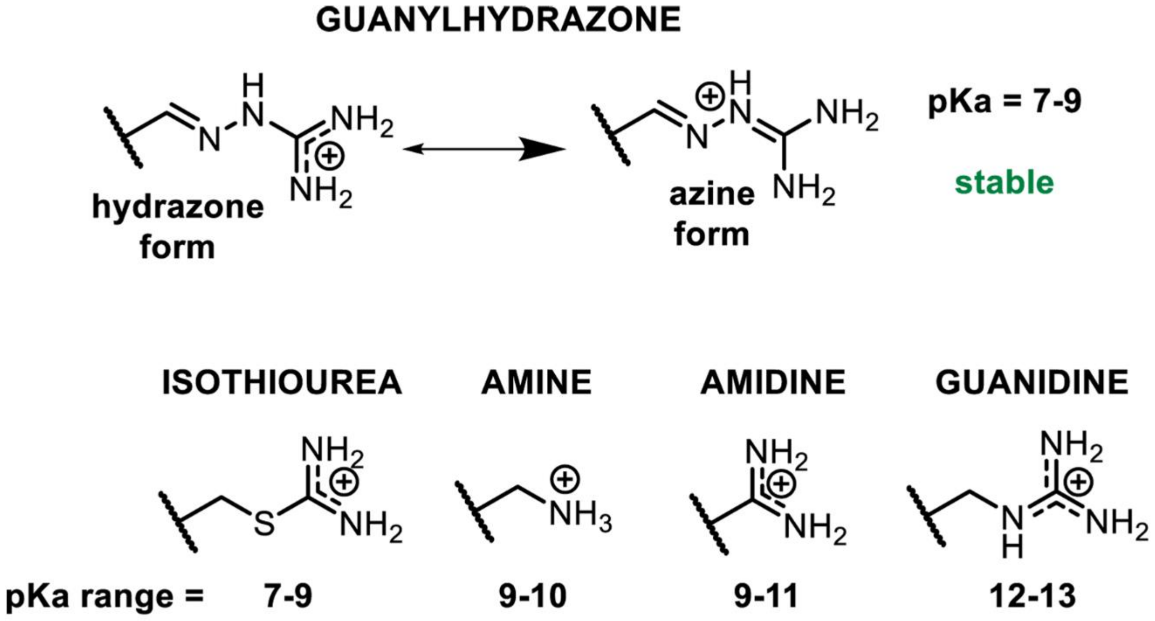

The figure shows positively charged N-containing groups indicating their pKa range

Supplementary Figure 4

Synthesis and analytical characterization of compound 2a

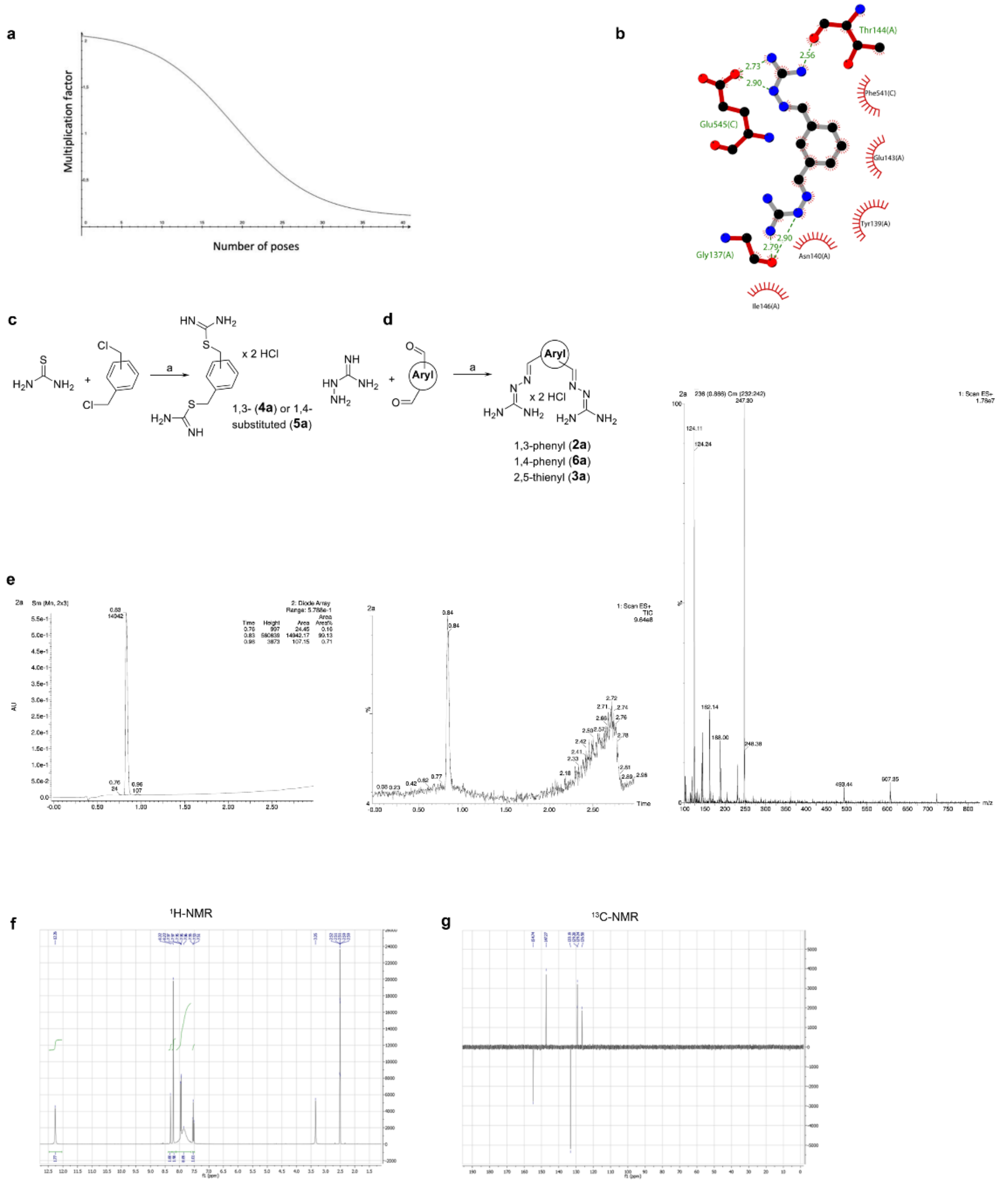

a, sigmoidal rectification function based on best pose recurrence in 40 independent runs; b shows the 2D interaction map of 2a. Ligand and protein atoms are represented in ball and stick, evidencing hydrogen bond distances (green lines) and hydrophobic interactions (red semicircles); c Isothioureas 4a and 5a were synthesized as dihydrochloride salts from the corresponding bis-chloromethyl benzenes and thiourea in good yields; d. bis-guanylhydrazones 2a, 3a and 6a were synthesized as dihydrochloride salts from the corresponding dialdehydes and aminoguanidine hydrochloride in good yields. e shows the diode Array LC trace (right panel), the TIC trace (middle panel), and an ES scan (left panel, 0.866 peak) for compound 2a; f and g show the ^{1}H-NMR and ^{13}C-NMR spectra of pure compound 2a, respectively.

Supplementary Figure 5

Compound 2a increases VPS26b and VPS29 in Neuro2a cells

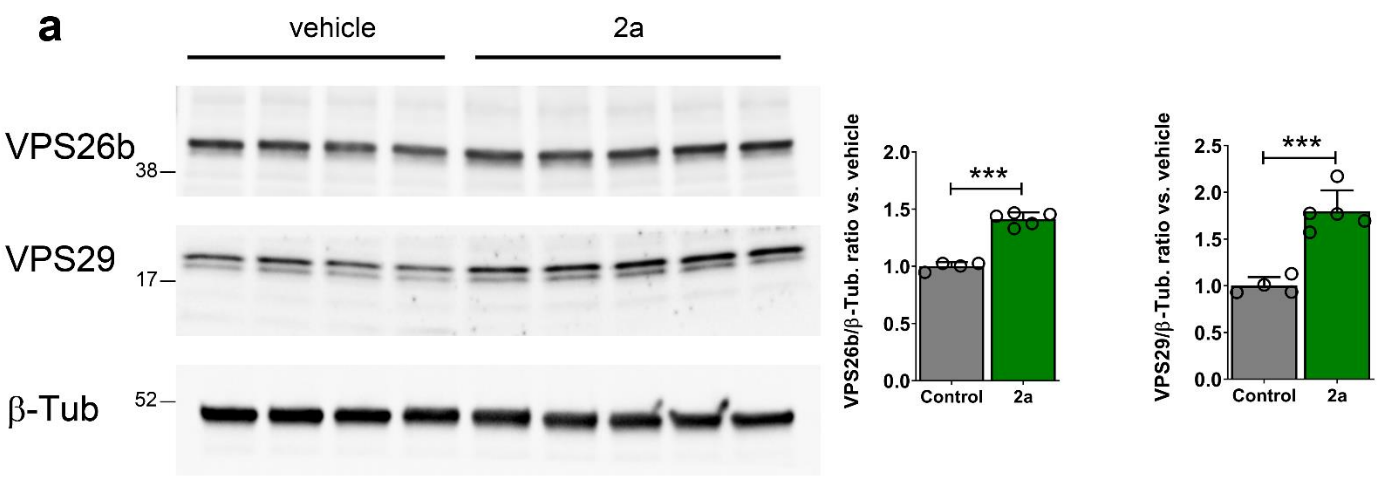

a, WBs for VPS26b, VPS29 and β-Tubulin in vehicle- and compound 2a-treated Neuro2a cells (10 µM, 48h, $3x10^5$ cells/well). Quantitative analyses of blots (means ± SD) are shown in histograms (control: n=4 independent wells, 2a: n=5 independent wells examined in one experiment, p<0.0001 for all the comparisons. Two-tailed Student's test was used to determine the statistical significance of data, *** p<0.0001

Supplementary Figure 6

Compound 2a stabilizes VPS35 levels in Neuro2a cells

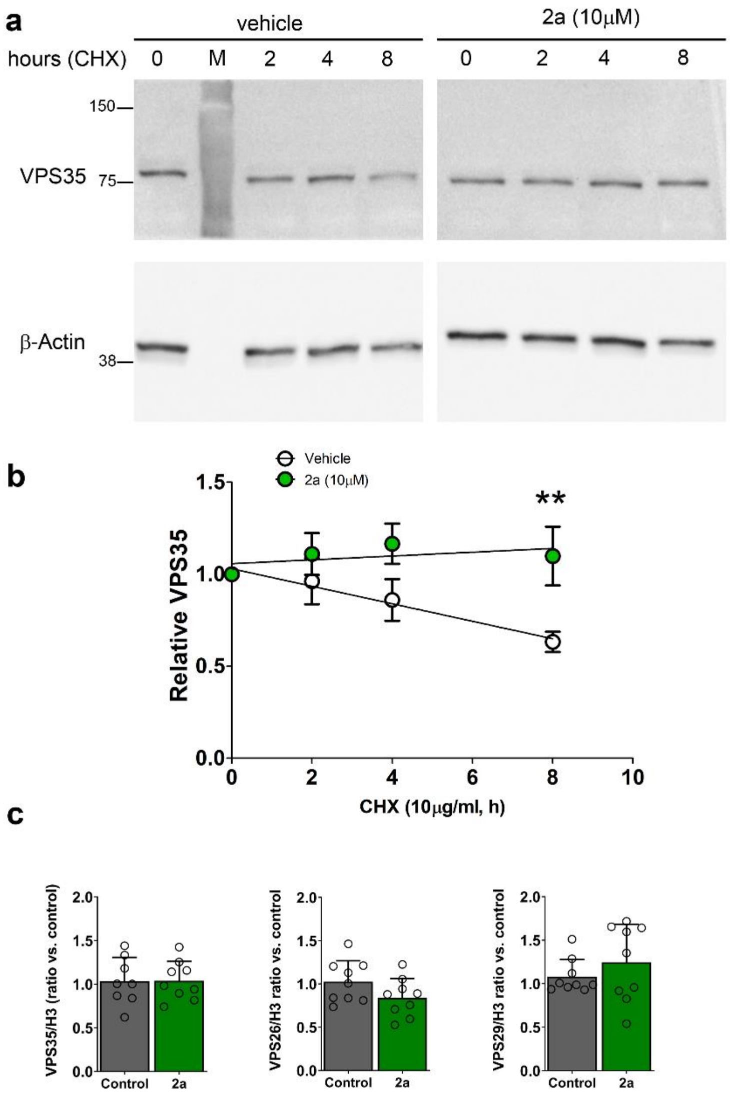

A cycloheximide (CHX) chase assay was performed to determine the degradation rate of VPS35 in Neuro2a cells (3x10^5 cells/well). Cells were treated with vehicle or 2a (10 µM) for 24 h, and then chased with CHX (10 µg/mL) for 2, 4 and 8h. Panel a shows representative WBs for VPS35 and β-Actin levels. Quantifications of normalized VPS35 levels (means normalized ratios ± SEM versus the time 0) are shown in panel b (vehicle: n=10 independent wells, 2a: n=8 independent wells obtained from 3 independent experiments; control vs. 2a at 8 hours: p=0.009). c, quantitative real time PCR for *Vps35*, *Vps26* and *Vps29* mRNA (Vps35: controls n=8 independent wells; 2a: n=9 independent wells; Vps26 and Vps29: n=9 independent wells/group); data were collected from 3 independent experiments. Compound-2a was incubated at 10 µM for 48h (3x10^5 cells/well); histograms report the ratio versus vehicle-treated cells (means ± SD). Two-way ANOVA followed by Bonferroni multiple comparisons test was used to analyze data plotted in panel b. Two-tailed Student's test was used to determine the statistical significance of data plotted in panel c. ** p<0.01.

Supplementary Figure 7

Compound 2a (10µM) does not perturb neuronal firing.

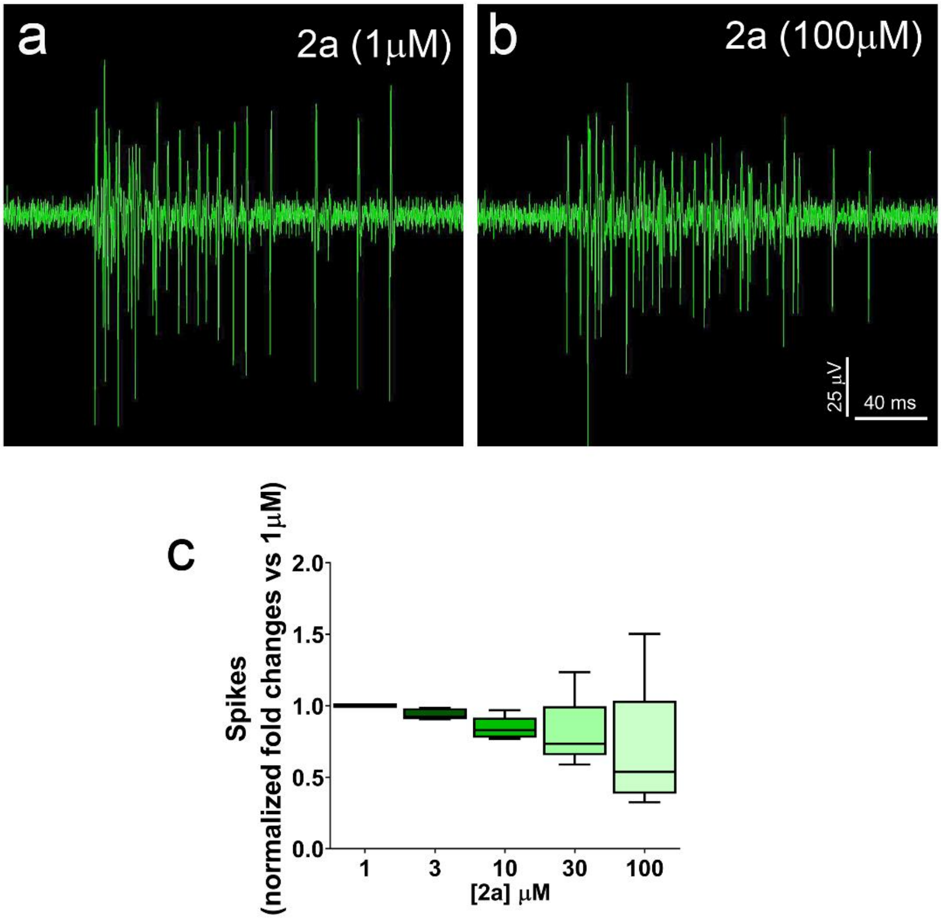

Panels a and b; neuronal activity from the same electrode of a representative MEA biochip ($3x10^5$ cortical neurons/chip, n=5 independent chips from 2 independent experiments) treated with 2a, at the concentration of 1 µM (a) and 100 µM (b). Neurons received increasing amounts of lead 2a (1, 3, 10, 30 and 100 µM) and were recorded every 5 min; c, quantifications of the firing activity reported as fold changes of spike numbers (box plots show median values; whiskers show 10-90 percentile) measured at the concentration of 1 µM. Kruskal-Wallis test followed by Dunn's multiple comparison test was used to analyze the data.

Supplementary Figure 8

Sh56 short interference sequence efficiently depletes VPS35.

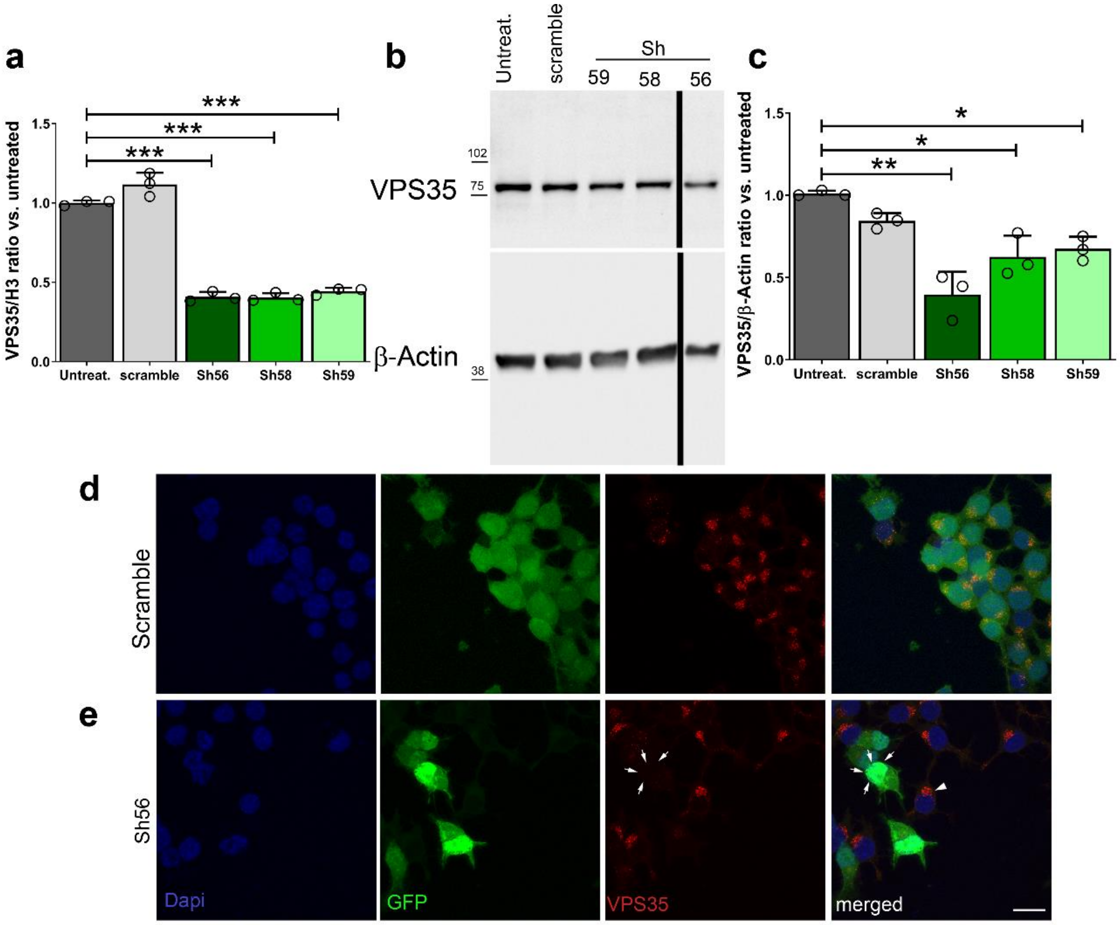

Neuro2a cells (3x10^5 cells/well) were transfected with scramble or with VPS35 RNAi plasmids (Sh56, Sh58 and Sh59, 2µg/well). Knock down was assessed by real time PCR analysis of RNAs sampled 48 h after the transfection. The histogram in panel a shows normalized *Vps35* mRNA levels (mean ± SD) reported as ratio versus untreated cells (n=3 independent wells/group from one experiment, p<0.0001 for all comparisons). WBs were carried out to verify VPS35 knockdown. Dividing black lines in panel b indicate cropped lanes from the same filter. Quantifications of normalized VPS35 protein levels (mean ± SD) reported as ratio versus untreated cells are shown in panel c (n=3 independent wells/group from 2 independent experiments, untreat. vs. sh56: p=0.006, untreat. vs. sh58: p=0.03, untreat. vs. sh59: p=0.02). Parallel cultures (3x10^5 cells/well) were transfected with pCAAG-GFP plasmids, along with scramble (d) or with Sh56 (e) plasmids and labelled for GFP and VPS35. Arrows in panel e show VPS35 immunoreactivity in a GFP$^+$ cells receiving Sh56 plasmids. The arrowhead in panel e indicates a GFP$^-$ cell form the same field that express VPS35 at normal levels (n=3 wells/group, data derived from 2 independent experiments). One-way ANOVA followed by Tukey's Multiple Comparison test was used to analyze data. * p<0.05, ** p<0.01, *** p<0.001. Scale bar 10 µm.

Supplementary Figure 9

Compound 2a reduces myelin clearance in G93A mice

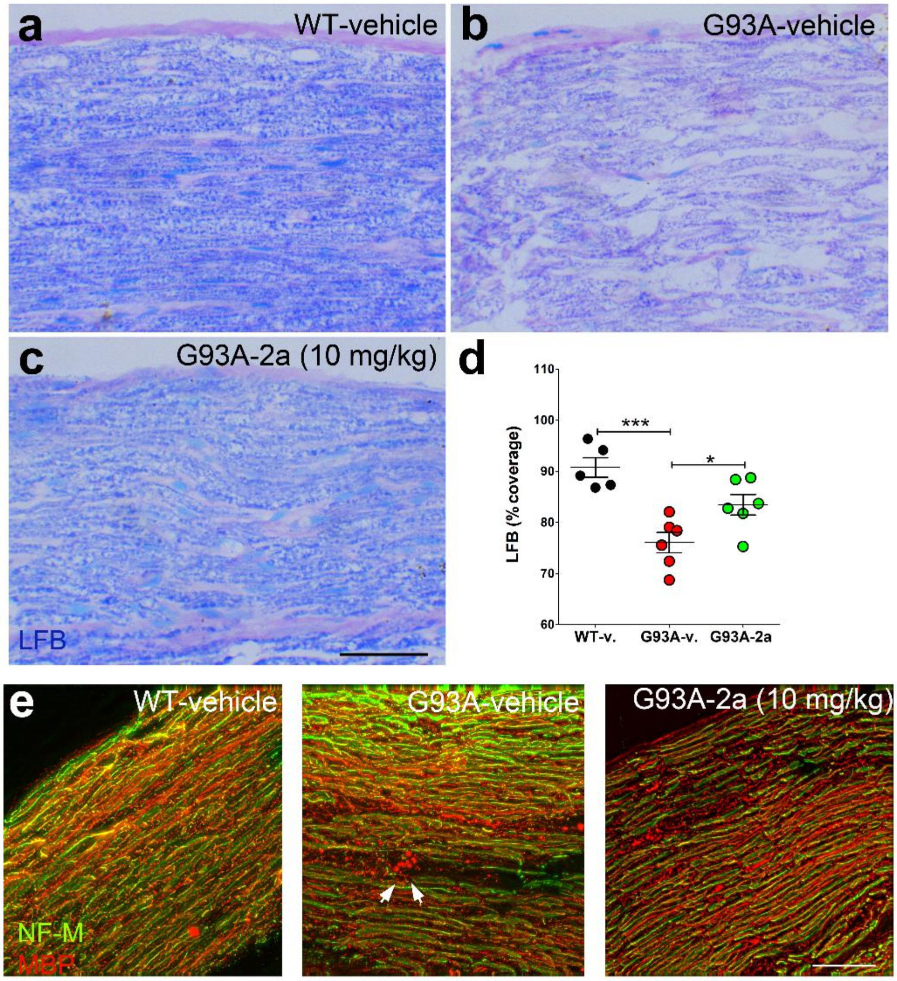

Panels a-c, Luxol Fast Blue (LFB) staining on sagittal sections of sciatic nerves from vehicle-treated WT mice (a), vehicle-treated G93A mice, (b) and compound 2a-treated G93A mice (c). Quantifications of (% ± SD) LFB covered areas are reported in panel d (WT-v: n=5 independent mice, G93A-v: n=6 independent mice; G93A-2a: n=6 independent mice, data are collected from 2 independent experiments), (WT-v vs G93A-v: p<0.0001, G93Av vs. G93A-2a: p=0.016). Parallel sections labelled for NF-M and MBP are shown in panel e (WT-v: n=5 independent mice, G93A-v: n=6 independent mice; G93A-2a: n=6 independent mice, data are collected from 2 independent experiments). Arrows in vehicle treated G93A mice show damaged myelin. One-way ANOVA followed by Tukey's Multiple Comparison test was used to analyze data of panels D. * p<0.05, *** p<0.001. Scale bar 50µm.

Supplementary Figure 10

Compound 2a does not modulate microglia/macrophage in G93A mice

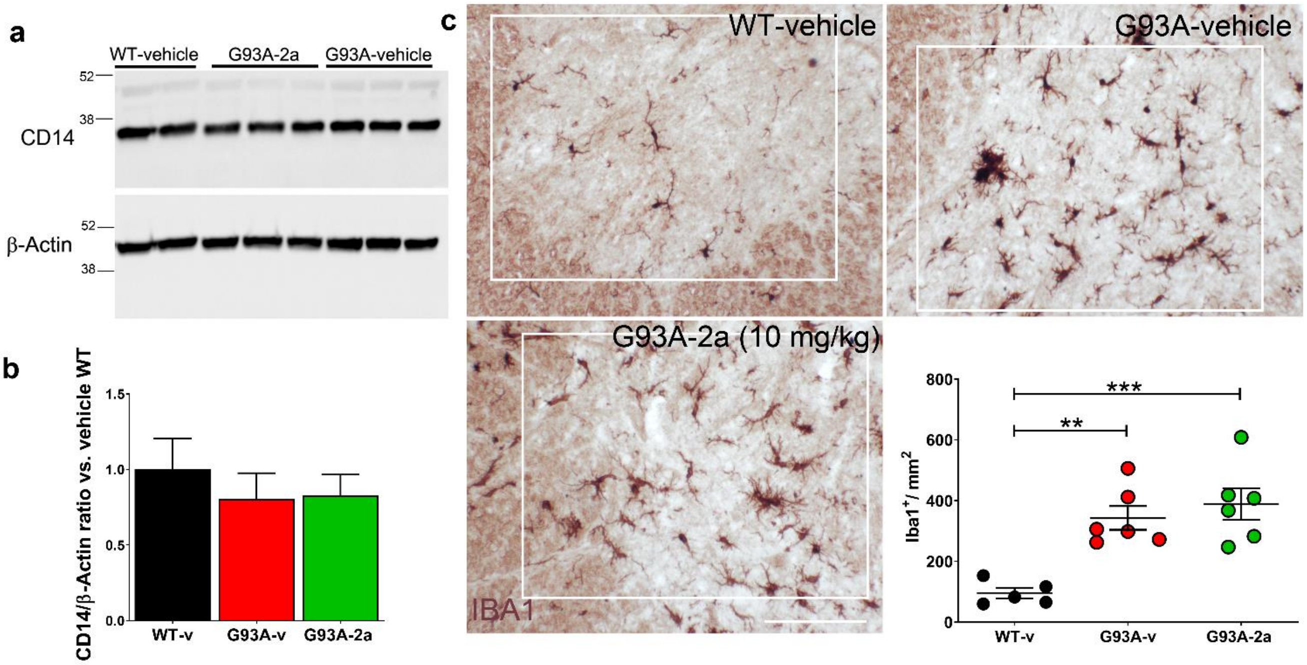

a shows a representative WB for CD14 in lumbar SCs protein extracts from vehicle-treated WT mice, vehicle-treated G93A mice and compound 2a (10 mg/kg)-treated G93A mice (sampled at day 100). Quantifications of normalized CD14 (mean ± SD) reported as ratio versus vehicle-treated WT mice are shown in b (n=3 independent mice/group from 2 WB experiments). Panel c shows IHC for Iba1 in vehicle-treated WT mice (n=5 independent mice), vehicle-treated G93A (n=6 independent mice) mice and compound 2a-treated G93A mice (n= 6 independent mice;10 mg/kg, day 100). Cell counts were done in a boxed region (320X260µm) that overlies the ventral horn of the SC (data derived from 2 independent experiments). The histogram in c shows quantifications (lines show means ± SEM, WT-v vs G93A-v: p=0.0019, WT-v vs. G93A-2a: p<0.0001). One-way ANOVA followed by Tukey's Multiple Comparison test was used to analyze data plotted in panel b and in panel c. ** p<0.01, *** p<0.001. Scale bar 50µm.

Supplementary Figure 11

Compound 2a increases VPS35 levels in G93A mice

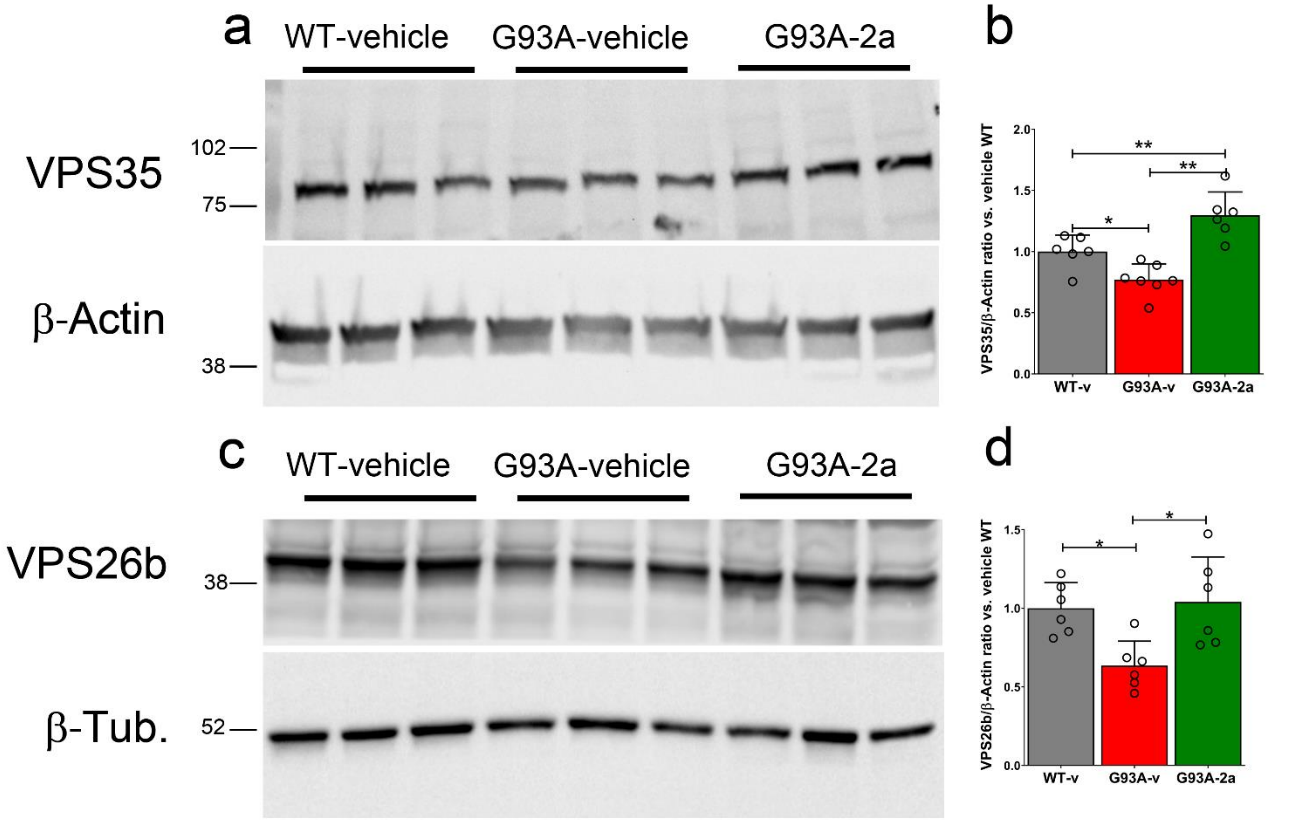

Panel a, representative WB for VPS35 and β-Actin in SCs from vehicle-treated WT mice, vehicle-treated G93A mice and compound 2a (10 mg/kg, sampled at day 100)-treated G93A mice (WT-v: n=6 independent mice, G93A-v: n=7 independent mice, G93A-2a: n=6 independent mice). Quantifications of normalized VPS35 levels reported as ratio versus vehicle-treated WT (mean ± SD) are shown in panel b (data are collected from 2 independent experiments; WT-v vs. G93A-v: p=0.015, G93A-v vs. G93A-2a: p=0.002, WT-v vs. G93A-2a: p=0.0038). Panel c, representative WB for VPS26b and β-Actin in SC lysates obtained from vehicle-treated WT mice, vehicle-treated G93A mice and lead 2a (10 mg/kg, sampled at day 100)-treated G93A mice (n=6 independent mice/group). Quantifications of normalized VPS26b levels reported as ratio versus vehicle-treated WT (mean ± SD) are shown in panel d (data are derived from 2 independent experiments, WT-v vs. G93A-v: p=0.03, G93A-v vs. G93A-2a: p=0.02). One-way ANOVA followed by Tukey's Multiple Comparison test was used to analyze data plotted in b and d. * p<0.05, ** p<0.01.

Supplementary Figure 12

VPS35 knockdown impairs lysosomal stability

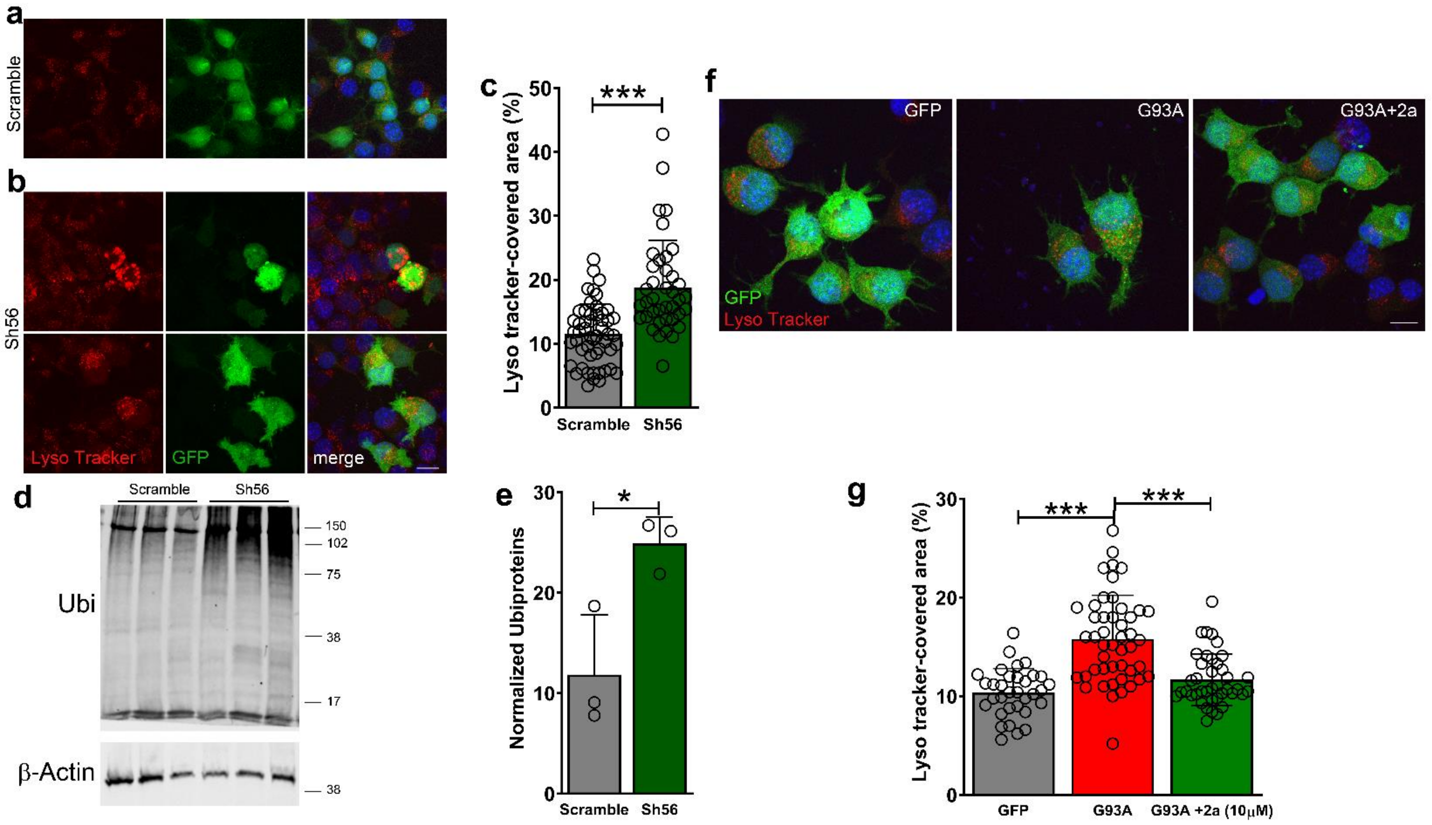

Panels a and b, Neuro2a cells (4x10^4cells/well) transfected with the scramble plasmid (0.5 μg/well, a) or with the VPS35 RNAi plasmids (Sh56, 0.5 μg/well, b) along with plasmid encoding the GFP (0.1 μg/well) and labelled with Lyso-tracker (75 nM). Quantifications of Lyso Tracker-covered areas (%± SD) in individual GFP$^+$ cells were done on merged confocal images and data are shown in c (scramble: n=52 cells, Sh56: n=39 cells, data are collected from 3 independent experiments, p<0.0001). Protein lysates from Neuro2a cells (3x10^5 cells/well) receiving scramble or Sh56 plasmids (2 μg/well for 48h) were labelled for Ubiquitin and β-Actin (d). The collective density of each Ubiquitinated-proteins lane was expressed relative to β-Actin levels and mean values ± SD are plotted in the histogram of panel e (n=3 independent well/group data are collected from 1 experiment, p=0.025). Neuro2a cells (4x10^4 cells/well) were transfected with plasmid encoding the GFP (0.6μg/well) or with the GFP (0.1 μg/well) and pcDNA3.1(+) SOD1^{G93A} (0.5μg/well) ± 2a (10μM) or vehicle. and 24 hours later they were starved and kept in FBS-free-medium ± 2a for additional 24 h. Cells were incubated with Lyso-tracker (75 nM). Panel f shows a representative merged confocal images of cells labelled for the GFP and the Lyso-tracker. Quantifications of Lyso-tracker-covered areas (%± SD) in each group are shown in panel g (GFP: n=33 cells, G93A: n=47 cells, G93A+2a: n=40 cells, data are collected from 3 independent experiments, p<0.0001 for all comparisons). Two-tailed Student's test was used to determine the statistical significance in panels c and e. One-way ANOVA followed by Tukey's Multiple Comparison test was used to analyze data plotted on panel g. * p<0.05; *** p<0.001. Scale bar 10μm.

Supplementary Figure 13

Compound 2a attenuates SOD1 aggregates in G93A mice

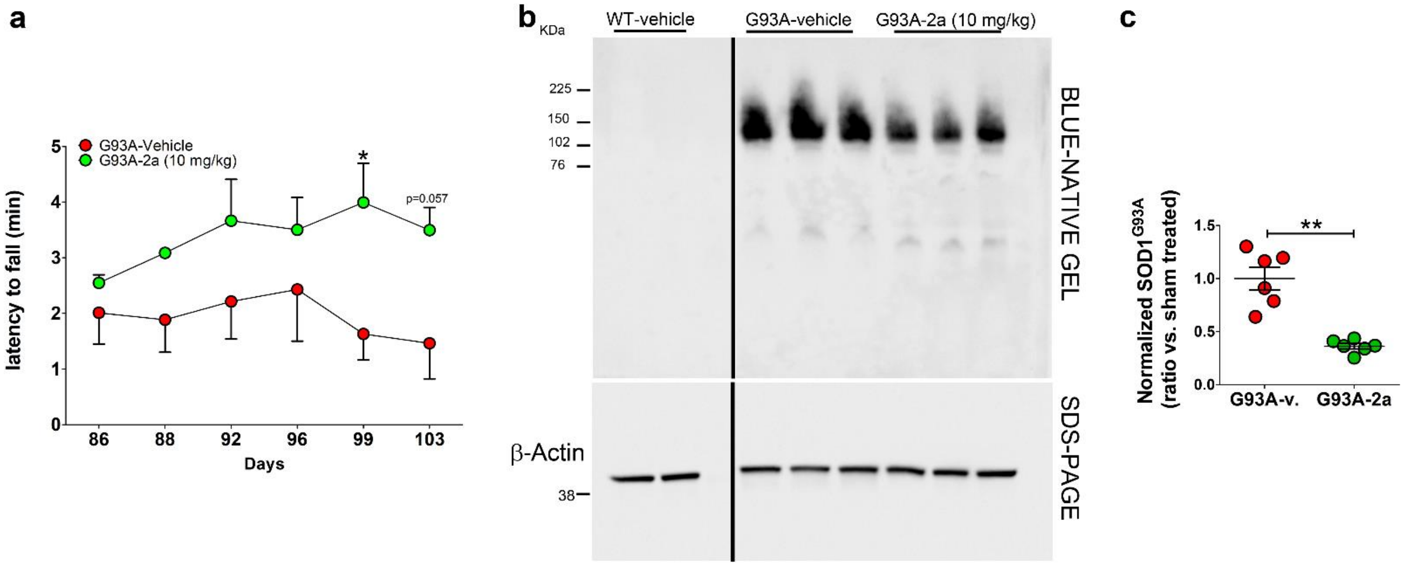

Panel a shows the latency to fall in vehicle- and compound 2a-treated G93A mice (10 mg/kg, n=4 independent mice/group, data are collected from 1 experiment). Data are reported as mean ± SD. A representative immuno-blot of a blue-native polyacrylamide gel loaded with lumbar SC extracts from vehicle- and 2a-treated mice is shown in b. Membranes were incubated in acetic acid to fix the proteins and probed with an anti-huSOD1 antibody. Equal amounts of protein extracts were loaded on parallel SDS-PAGE, blotted on nitrocellulose filters and probed with an anti β-Actin antibody. Dividing black lines mark lanes cropped from the same filter; c, quantifications of the mutant forms of the SOD1 from mice treated with sham or with compound 2a (G93A-v: n=6 independent mice, G93A-2a: n=6 independent mice, data are derived from 2 independent experiments). Two-tailed Student's test was used to determine the statistical significance in a ($p=0.011$); the Mann Whitney test was used to determine statistical significance in c ($p=0.0022$). * $p<0.05$, ** $p<0.01$

Supplementary Figure 14

Characterization of iPSC lines

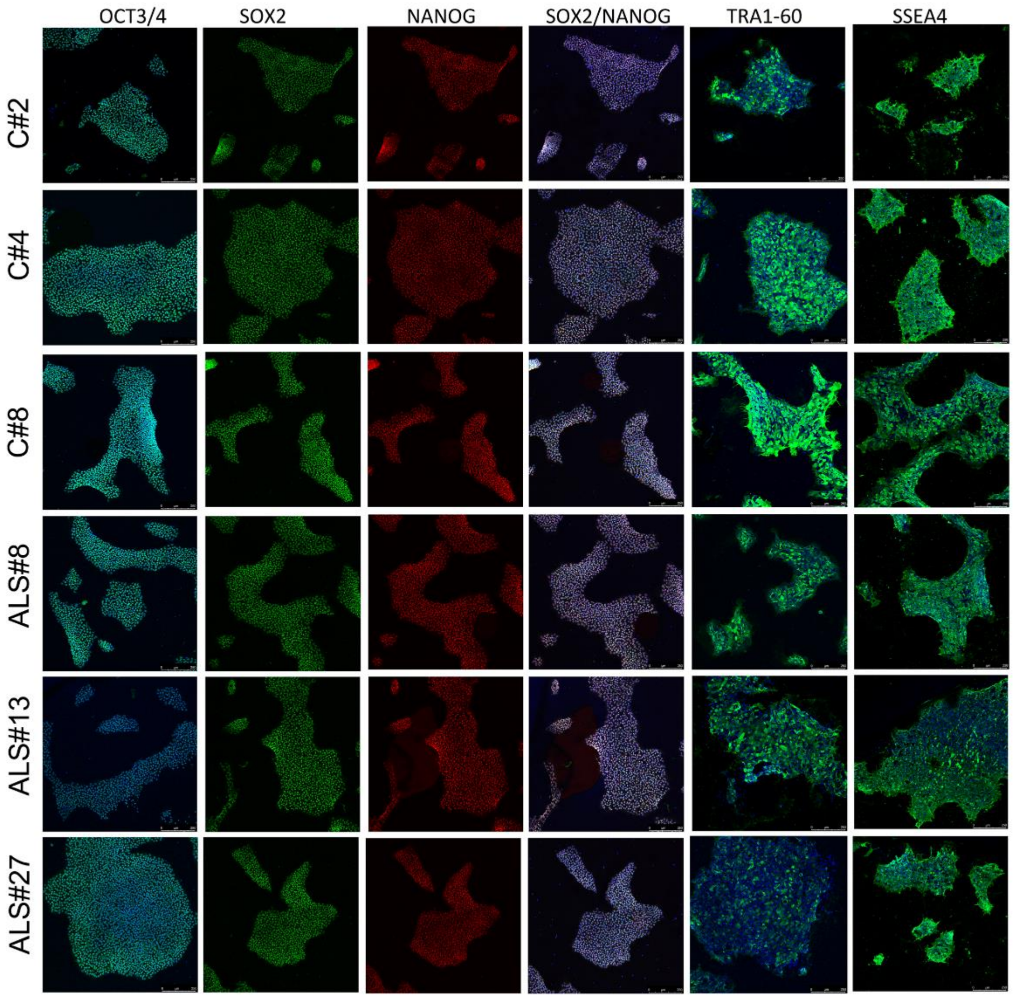

IF analysis of iPSC cell lines for the following pluripotency markers: OCT4, SOX2, NANOG, TRA1-60, SSEA4. Data are derived from 1 experiment. Scale bar 250 µm.

Supplementary Figure 15

Karyotype and MN differentiation of iPSC lines

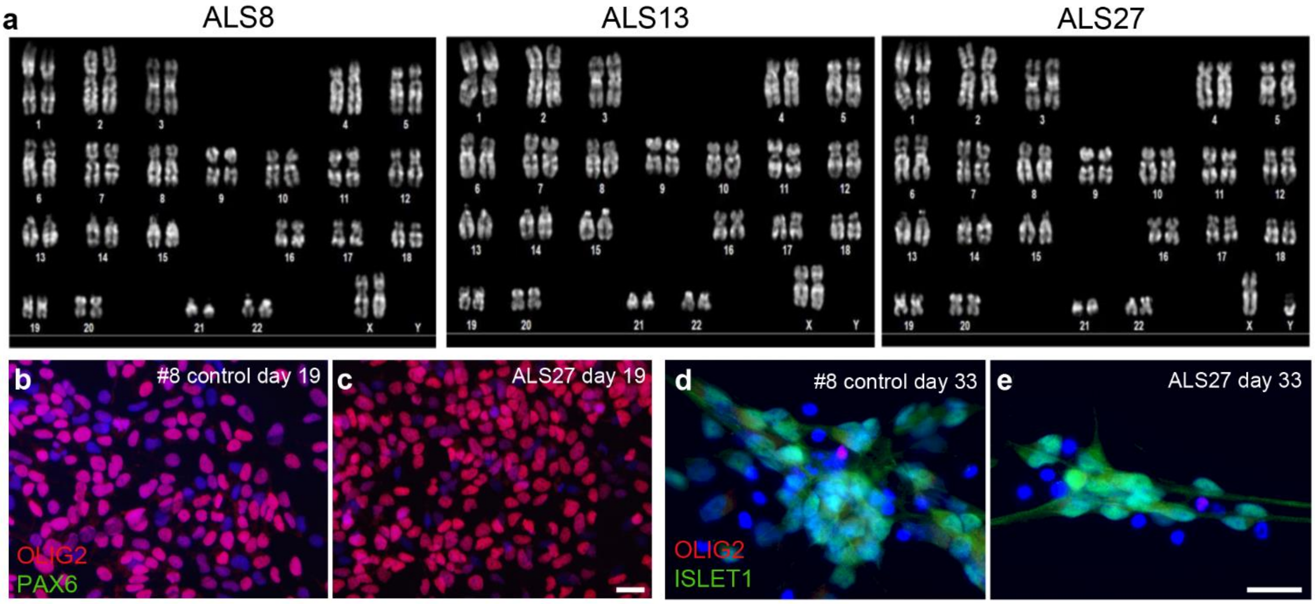

Panel a, high –resolution analysis of karyotypes of ALS iPSC lines indicating a normal chromosomal content (data are derived from one experiment). Panel b and c, representative IF for OLIG2 and PAX6 in control (b) and ALS (c) cultures collected 19 days after the induction of the MN differentiation protocol (data are representative of 3 independent experiments). Panel d and e, representative IF for OLIG2 and ISLET1 in control (d) and ALS (e) cultures at the end of the MN differentiation protocol (data are representative of 3 independent experiments). Scale bar 20 µm.
