# Supplementary material for: Retromer stabilization results in neuroprotection in a model of Amyotrophic Lateral Sclerosis
Source: Nat Commun. 2020 Jul 31;11:3848. doi: 10.1038/s41467-020-17524-7 (PMC7395176; doi:10.1038/s41467-020-17524-7)
Supplement: Supplementary file 2 — Reporting Summary [file 41467_2020_17524_MOESM2_ESM.pdf]

nature research

Corresponding author(s): Luca Muzio

Last updated by author(s): Jun 22, 2020

Reporting Summary

Nature Research wishes to improve the reproducibility of the work that we publish. This form provides structure for consistency and transparency in reporting. For further information on Nature Research policies, seeAuthors & Referees and theEditorial Policy Checklist .

Statistics

For all statistical analyses, confirm that the following items are present in the figure legend, table legend, main text, or Methods section.

n/a	Confirmed	
☐	☒	The exact sample size (*n*) for each experimental group/condition, given as a discrete number and unit of measurement
☐	☒	A statement on whether measurements were taken from distinct samples or whether the same sample was measured repeatedly
☐	☒	The statistical test(s) used AND whether they are one- or two-sided<br>*Only common tests should be described solely by name; describe more complex techniques in the Methods section.*
☒	☐	A description of all covariates tested
☐	☒	A description of any assumptions or corrections, such as tests of normality and adjustment for multiple comparisons
☐	☒	A full description of the statistical parameters including central tendency (e.g. means) or other basic estimates (e.g. regression coefficient) AND variation (e.g. standard deviation) or associated estimates of uncertainty (e.g. confidence intervals)
☐	☒	For null hypothesis testing, the test statistic (e.g. *F*, *t*, *r*) with confidence intervals, effect sizes, degrees of freedom and *P* value noted<br>*Give P values as exact values whenever suitable.*
☒	☐	For Bayesian analysis, information on the choice of priors and Markov chain Monte Carlo settings
☒	☐	For hierarchical and complex designs, identification of the appropriate level for tests and full reporting of outcomes
☒	☐	Estimates of effect sizes (e.g. Cohen's *d*, Pearson's *r*), indicating how they were calculated

Our web collection on statistics for biologists contains articles on many of the points above.

Software and code

Policy information about availability of computer code

Data collection	NHI-Image J (1.52t) software (US National Institutes of Health)<br>AutoDock4.2 software (The Script Research Institute)<br>Marvin Sketch v20.13.0 (Chemaxon packages; https://chemaxon.com/)<br>Python Molecular Viewer (http://mgltools.scripps.edu)<br>MC-Rack Software version 4.6.2(MCS GmbH)<br>Leica Application Suite X 2.0.0.14332 (Leica)<br>Image Lab 6.0.1 built 34 (Bio_Rad Laboratories Inc.)<br>TOPSPIN 4.0.5 software (Bruker)<br>MultiQuant 2.1 software (SCIEX)<br>GEN5 2.03.1 (Agilent)<br>Wallac 1420 (Perkin Elmer)<br>LightCycler software (Roche)
Data analysis	PRISM5.01 (GraphPad Software, La Jolla, CA, USA)<br>PRISM8.4.2 GraphPad Software, La Jolla, CA, USA)<br>BioVinci 1.1.5, r20181005 (BioTuring Inc.)

For manuscripts utilizing custom algorithms or software that are central to the research but not yet described in published literature, software must be made available to editors/reviewers. We strongly encourage code deposition in a community repository (e.g. GitHub). See the Nature Research guidelines for submitting code & software for further information.

Data

Policy information about availability of data

All manuscripts must include a data availability statement. This statement should provide the following information, where applicable:

- Accession codes, unique identifiers, or web links for publicly available datasets
- A list of figures that have associated raw data
- A description of any restrictions on data availability

The data that support the findings of this study are available from the corresponding author (luca.muzio@hsr.it), upon reasonable request. The authors declare that the data supporting the findings of this study are available within the paper and its supplementary information files.

Field-specific reporting

Please select the one below that is the best fit for your research. If you are not sure, read the appropriate sections before making your selection.

[x] Life sciences   [ ] Behavioural & social sciences   [ ] Ecological, evolutionary & environmental sciences

For a reference copy of the document with all sections, see nature.com/documents/nr-reporting-summary-flat.pdf

Life sciences study design

All studies must disclose on these points even when the disclosure is negative.

Sample size	The number of G93A mice used in pharmacological experiments were calculated according our previously published experiments (Rossi et al., Cell Death Dis. 2018). The exact sample size for each experimental group was provided in the manuscript
Data exclusions	No data were exclude from the study
Replication	Data were successfully replicated. In vivo studies involving G93A mice and compound 2a were done on two independent cohorts of mice purchased from Jackson laboratories. PK experiments were done on two cohorts of mice purchased from Charles River Italy (C57BL6/J, WT). Experiments involving IF, WBs, IHC and real time PCR were done in a minimum of 3 up to 9 independent mice. Experiments involving cultured cells were performed on a minimum of 3 up to 24 independent samples. Key in vitro experiments were replicated independently to confirm robustness of major findings
Randomization	Sod1 transgenic mice were randomly allocated in sham- or compound 2a-treated groups. The pharmacological treatment started at day 30. At this time point ALS mice did not show any sign of the disease and therefore they were randomly distributed in control and experimental groups. Cell based experiments involving Neuro2a or iPSCs-derived MNs were done as follow: cells were maintained in vitro, divided equally to each group and then treated with drug agents.
Blinding	Reconstitution of the compound 2a powder for in vivo experiments was performed by colleagues from outside the team, who took care of blinding the treatment to operators of our laboratory. Therefore, injections and manipulation of WT and Sod1 mice were done by operators blind to the treatment. In vitro treatments were done according standard procedures and blinding was not used. Imaging experiments (quantitative analyses) of cells or tissue sections were manually blinded before the analysis.

Reporting for specific materials, systems and methods

We require information from authors about some types of materials, experimental systems and methods used in many studies. Here, indicate whether each material, system or method listed is relevant to your study. If you are not sure if a list item applies to your research, read the appropriate section before selecting a response.

Materials & experimental systems

n/a	Involved in the study	
[ ]	[x]	Antibodies
[ ]	[x]	Eukaryotic cell lines
[x]	[ ]	Palaeontology
[ ]	[x]	Animals and other organisms
[ ]	[x]	Human research participants
[x]	[ ]	Clinical data

Methods

n/a	Involved in the study	
[x]	[ ]	ChIP-seq
[x]	[ ]	Flow cytometry
[x]	[ ]	MRI-based neuroimaging

Antibodies

Antibodies used	A)rabbit α-VPS26 1:1000 (ab23892 Abcam) B)goat α-VPS35 1:800 (ab10099 Abcam), C) rabbit α-VPS35 1:1000 (ab97545 Abcam), D)rabbit α-VPS26b 1:1000 (15915-1-AP, ProteinTech), E)rabbit α-VPS29 1:1000 (ab236796 Abcam), F)rabbit α-Iba1 1:500 (Wako), G)goat α-ChAT 1:200 (Millipore ab144p), H)mouse α-NeuN 1:800 (Millipore MAB377), I)mouse α-Golgin97 1:700 (Thermofisher

A21270), J)mouse α-βActin 1:25000 (Sigma), K)Chicken α-Neurofilament-medium 1:500 (Biolegend PCK-593P), L)rat α-MBP 1:100 (kindly provided by Dr. A. Bolino), M)mouse α-CI-MPR 1:100 (Novus bio. NB-300-514), N)rat α-CTSD 1:100 (R&D system MAB1029), O)mouse α-Ubiquitin 1:500 (Millipore MAB1510), P)mouse α-GM130 1:100 (BD biosciences, 610823), Q)Rabbit α-SOD1 (1:2000, Genetex), R)mouse α-SOX2 1:100 (R&D, MAB2018), S) goat α-NANOG 1:200 (R&D, AF1997), T)mouse α-OCT3-4 1:500 (Santa Cruz, sc-5279); U) mouse α-SSEA4 1:400 (Millipore MAB4304), V)mouse α-TRA1-60 1:200 (Millpore MAB4360), X) mouse α-b-Tubulin 1:5000 (Immunological Sciences MAB-80143), Y) rabbit α-CD14 1:1000 (Bioss Inc)

Validation

A) Host: rb cat. Code: ab23892 lot.:gr3176799/2 validation: abcam validated using KO cell lines reactivity :Mouse, Rat, Human guarantee: Abpromise®guarantee
B) Host: goat cat.code: ab10099 lot.: GR271565-24 validation: abcam validated use for WB, ICC/IF reactivity: Mouse, Human guarantee: Abpromise® guarantee
C) Host: rb cat Code: ab97545 lott.: GR254231-11 validation: abcam WB reactivity: Human, Mouse, Rat, Cow guarantee: Abpromise® guarantee
D) Host: rb cat. Code: 15915-1-ap lott.:080159 validation: proteintech WB, IP, IF, ELISA reactivity: human, mouse, rat guarantee: Proteintech Guarantee
E) Host: rb cat.code: ab98929 lott.:GR3267980-7 validation: abcam WB reactivity: Human,Mouse, Rat, Rabbit, Horse, Chicken, Guinea pig, Cow, Cat, Dog, Drosophila melanogaster, Zebrafish guarantee: Abpromise® guarantee
F) Host: rb cat code: 019-19741 lott.: WDK2121 validation: wako IHC, ICC, FACS reactivity: Human, Mouse, Rat
G) Host: gt cat code: ab144p Lot # 2464504 validation: Millipore IH(P), ICC, IHC, WB reactivity: H, R, M, Mk, Op, Av, Ch, Gp, Zebrafish guarantee: Quality Control Testing Routinely evaluated by Western Blot on mouse brain lysates.
H) Host: ms cat code: MAB377 lott.:3045564 validation: millipore WB, IC, IH(P) reactivity: Av, Ch, Ft, H, M, Po, R, Pm, Sal guarantee: Routinely evaluated by immunohistochemistry on brain tissue.
I) Host: ms cat.code: A-2127 0 lott.:1806354 validation: thermofisher ICC, IP, WB, IF, IHC, IM reactivity: Dog, Human, Mouse, Non-human primate guarantemofisher Performance Guarantee
J) Host: ms cat.code: A 1978 lott.: 22190701 validation: sigma WB, IHC reactivity: pig, Hirudo medicinalis, bovine, rat, canine, feline, human, rabbit, carp, mouse, guinea pig, chicken, sheep
K) Host: ch cat.code: PCK-593p lott. Poly28227 validation: BioLegend WB, IF reactivity: Human, Rat, Mouse, Cat guarantee: control tested by Western blotting.
L) Host: rat Custom Antibody, validated in Bolino et al., EMBO molecular Medicine 2016.
M) Host: ms cat.code: NB300-514 lott: 080713 validation: NOVUSBIO Western Blot, ELISA, Flow Cytometry, Immunocytochemistry/Immunofluorescence, Immunohistochemistry, Immunohistochemistry-Paraffin, Immunoprecipitation, CyTOF-ready reactivity: Hu, Mu, Rt, Bv,Pm
N) Host: rat cat.code: mab1029 lott.: ISH0213121 validation: RD system wb reactivity: ms guarantee: authorized by ou Quality Assurance program ReD
O) Host: ms cat.code:MAB1510lott.:3091712 validation: MILLIPORE WB, EM, IH(P), IP reactivity: B, Ch, Dr, H, M, R guarantee: Quality Control Testing Routinely evaluated by Western Blot on Jurkat
P) Host: ms cat code: 610823 lott.: 7163670 validation:BD bioscences wb,ip ractivity: rat, humann, dog, mause guarantee: Quality Control Testing Routinely evaluated by Western Blot
Q) Host:rb cat.code: gtx100554 validation: genetex WB, ICC/IF, IHC-P, ELISA, sELISA reactivity: Human, Mouse, Rat, Alligator, Bird guarantee: ko validation-orthogonal validation
R) Host: Monoclonal Mouse IgG2A Clone # 245610, validation: human, mouse, and rat SOX2 in Western blots, reactivity: Human, Mouse, Rat
S) Host: Polyclonal Goat IgG, validation: Nanog in BG01V Human Stem Cells, reactivity: human
T) Host: mouse, validation: western blot of F9, ES-D3 and NTERA-2 cl.D1 cells, reactivity: mouse, rat e human
U) Host: mouse, validation: pluripotent human embryonic stem (ES) cells. reactivity: human and mouse
V) Host: mouse, validation: NTERA-2 cl.D1 whole cell lysate (pluripotent stem cells derived from teratocarcinoma and are considered the malignant counterparts of human embryonic stem cells), reactivity: human
X) Host: mouse, validation Immunological Sciences WB, reactivity: Human, Mouse, Rat
Y) Host: rabbit, validation: WBs by Bioss Inc, reactivity: Human,Rat,Mouse

Eukaryotic cell lines

Policy information about cell lines

Cell line source(s)

Neuro2a cell line were purchased from ATCC (ATCC® CCL-131™)
We generated iPSCs lines from primary fibroblasts. Fibroblasts were obtained from skin biopsies from ALS patients and healthy volunteers.

Authentication

Neuro2a cell line did not receive authentication.
We generated iPSCs lines from healthy subject and ALS patients according standard procedures. ALS iPSCs cell lines received karyotyping analysis by ISENET Biobanking service unit in Milan. However, they were not authenticated

Mycoplasma contamination	Cells used in this study were routinely assayed for mycoplasm by PCR (details about the method are available in the source data file) and all cell lines tested negative for mycoplasma contamination .
Commonly misidentified lines (See ICLAC register)	These cell lines are not commonly misidentified

Animals and other organisms

Policy information about studies involving animals; ARRIVE guidelines recommended for reporting animal research

Laboratory animals	Transgenic mutant SOD1 mice carrying the SOD1G93A allele (strain B6SJL-TgN[SOD1-G93A]1GUR) from Jackson laboratories, males, treatment: from day 30 to day 100.<br>Wild type mice (strain C57BL/6J), used for breeding and experimental purposes, from Charles River (Italy), males and females (only breeding), treatments: from 2 months.<br>Both transgenic and WT mice had food and water freely available in the home-cage. The holding room was on a 12-h light–dark cycle, the temperature of the room was 22±0.2 °C.
Wild animals	This study did not involve wild animals
Field-collected samples	This study did not involve samples collected from the field
Ethics oversight	Mice were maintained under pathogen-free conditions at San Raffaele Hospital mouse facility (Milan, Italy), in accordance with the European Communities Council Directive of 24 November 1986 (86/609/EEC). The Ethics Review Committee approved experimental protocols according guidelines from the Italian Ministry of Health and from the Institutional Animal Care and Use Committee of the San Raffaele Scientific Institute (protocol number 704/2015PR).

Note that full information on the approval of the study protocol must also be provided in the manuscript.

Human research participants

Policy information about studies involving human research participants

Population characteristics	Skin biopsies from 3 ALS patients (age: 45-55; sex: one male and two females) and 3 healthy volunteers (age: 35-60; females).
Recruitment	A skin punch biopsy was performed and dermal fibroblasts were acquired from this biopsy under informed consent as outlined and approved by Ospedale San Raffaele (Milan, Italy). We performed the genotype of ALS patients and only patients carrying mutation in SOD1 gene were include in the study. To minimize the potential bias deriving from the limited number of controls- and patients-derived iPSC lines dedicated to the study, we provided the generation/characterization of control and ASL iPSCs lines and their differentiation to MNs in the same experimental setting. The efficiency of these protocols and major outcomes were assessed at intermediate time points following literature precedents (Du, Z. W. et al Nat Commun 6, 6626, 2015).
Ethics oversight	Approved by local ethics committee (Ospedale San Raffaele): BANCA INSPE/8-10-19 and MND Genotipo Fenotipo/16-5-19.<br>Paraffin embedded SCs were obtained from ALS patients and non-neurological controls from the Target ALS Human Postmortem Tissue Core. Sections were stored at the INSPE tissue Bank (San Raffaele Scientific Institute).

Note that full information on the approval of the study protocol must also be provided in the manuscript.
